# Housing environment and early childhood development in sub-Saharan Africa: A cross-sectional analysis

**DOI:** 10.1371/journal.pmed.1003578

**Published:** 2021-04-19

**Authors:** Yaqing Gao, Long Zhang, Ashish Kc, Yinping Wang, Siyu Zou, Chunyi Chen, Yue Huang, Xiaoyi Mi, Hong Zhou

**Affiliations:** 1 Department of Maternal and Child Health, School of Public Health/National Health Commission Key Laboratory of Reproductive Health, Peking University, Beijing, China; 2 University of Michigan School of Dentistry, Ann Arbor, Michigan, United States of America; 3 International Maternal and Child Health, Department of Women’s and Children’s Health, Uppsala University, Uppsala, Sweden; London School of Hygiene and Tropical Medicine, UNITED KINGDOM

## Abstract

**Background:**

The influence of the safety and security of environments on early childhood development (ECD) has been under-explored. Although housing might be linked to ECD by affecting a child’s health and a parent’s ability to provide adequate care, only a few studies have examined this factor. We hypothesized that housing environment is associated with ECD in sub-Saharan Africa (SSA).

**Methods and findings:**

From 92,433 children aged 36 to 59 months who participated in Multiple Indicator Cluster Survey (MICS) in 20 SSA countries, 88,271 were tested for cognitive and social–emotional development using the Early Childhood Development Index (ECDI) questionnaire and were thus included in this cross-sectional analysis. Children’s mean age was 47.2 months, and 49.8% were girls. Children were considered developmentally on track in a certain domain if they failed no more than 1 ECDI item in that domain. In each country, we used conditional logistic regression models to estimate the association between improved housing (housing with finished building materials, improved drinking water, improved sanitation facilities, and sufficient living area) and children’s cognitive and social–emotional development, accounting for contextual effects and socioeconomic factors. Estimates from each country were pooled using random-effects meta-analyses. Subgroup analyses were conducted by the child’s gender, maternal education, and household wealth quintiles. On-track cognitive development was associated with improved housing (odds ratio [OR] = 1.15, 95% CI 1.06 to 1.24, *p* < 0.001), improved drinking water (OR = 1.07, 95% CI 1.00 to 1.14, *p* = 0.046), improved sanitation facilities (OR = 1.15, 95% CI 1.03 to 1.28, *p* = 0.014), and sufficient living area (OR = 1.06, 95% CI 1.01 to 1.10, *p* = 0.018). On-track social–emotional development was associated with improved housing only in girls (OR = 1.14, 95% CI 1.04 to 1.25, *p* = 0.006). The main limitations of this study included the cross-sectional nature of the datasets and the use of the ECDI, which lacks sensitivity to measure ECD outcomes.

**Conclusions:**

In this study, we observed that improved housing was associated with on-track cognitive development and with on-track social–emotional development in girls. These findings suggest that housing improvement in SSA may be associated not only with benefits for children’s physical health but also with broader aspects of healthy child development.

## Abstract 2

In a cross-sectional analysis of nationally representative survey data from 20 countries, Yaqing Gao and colleagues investigate associations between housing environment and cognitive and social development in children.

## Author summary

**Why was this study done?:**

- Sub-Saharan Africa (SSA) had the largest number of children (29.4 million) with low cognitive and/or social–emotional development worldwide in 2010.
- Housing might be linked to early childhood development (ECD) by affecting a child’s health and a parent’s ability to provide adequate care but is neglected in public health, especially in low- and middle-income countries (LMIC).

**What did the researchers do and find?:**

- We tested the hypothesis that improved housing is associated with on-track development in 2 ECD domains (cognitive and social–emotional).
- We analyzed data from 88,271 children aged 36 to 59 months in 20 SSA countries collected from nationally representative surveys conducted between 2010 and 2019.
- Improved housing was associated with 15% higher odds of being developmentally on track in the cognitive domain. Overall, improved housing was associated with on-track social–emotional development in girls but not boys.

**What do these findings mean?:**

- Housing improvement in SSA may be associated not only with benefits for children’s physical health but also with broader aspects of healthy child development.
- Future work to establish a causal link between housing and ECD and to assess its underlying mechanisms is crucial for the designing of cost-effective ECD interventions in SSA.

## Introduction

Early childhood development (ECD) refers to the development of core age-appropriate perceptual, physical, cognitive, social–emotional, and language skills [1], which influences individual academic, behavioral, and economic accomplishments [2]. In 2010, an estimated 80.8 million children in low- and middle-income countries (LMIC), aged 36 to 59 months, were not on track for cognitive and/or social–emotional development [3]. The Nurturing Care Framework demonstrated that, to reach their full developmental potential, children need 5 components of nurturing care: good health, adequate nutrition, responsive caregiving, opportunities for learning, and safety and security [4]. Most ECD research investigating risk factors and interventions focused on nutritional supplementation, responsive care, and learning opportunities [5], yet the safety and security of children’s environments has received little attention.

There is a lack of research on the influence of housing, as a central pillar of human security, on ECD. The hypothesized mechanisms of the association between housing and ECD are as follows. First, improved housing quality can reduce the exposure to biological (e.g., mold and pests), physical (e.g., noise, extreme temperatures, and poor ventilation), and chemical hazards (e.g., lead and arsenic) [6], which might result in negative health consequences for children [7]. Cross-sectional studies conducted in sub-Saharan Africa (SSA) showed that children living in improved housing (defined as housing with improved drinking water and sanitation facilities, sufficient living area, and finished building materials) had a lower likelihood of malaria, stunting, diarrhea, and anemia [8,9]. Improved physical health, in turn, may benefit the developing brain [10]. Second, a longitudinal study in the United States showed that children living in homes with more rooms and higher levels of cleanliness and safety experienced less physiological stress [11], which might improve their emotional stability and learning [12]. Third, improved housing quality, such as less dampness and warmth, may also reduce mental health problems and stress in parents [13], promoting their ability to engage in stimulating activities with their children. A longitudinal study in the US indicated that mothers’ psychological distress and parenting stress were important mediators in the associations between poor housing quality and children’s internalizing and externalizing problems [12]. Research on the direct associations between housing quality and ECD has been limited and mostly conducted in the US and Europe [7,14]. For example, a longitudinal study carried out in the US showed that children living in a house with environmental deficiencies such as a leaking roof, broken windows, and rodents had lower average reading and math skills [12]. The association between housing and ECD in LMIC, which have more severe housing and ECD problems than high-income countries, remains uncertain.

Africa has the highest rate of population growth and the most acute need for better housing worldwide [15]. The unprecedented modernization and economic growth in Africa have provided a significant opportunity to build healthier homes. Indeed, the prevalence of improved housing doubled in SSA from 2000 to 2015 [16]. However, an unacceptably large proportion of individuals still live in unimproved housing [16]. SSA had the largest number of children (29.4 million) experiencing low cognitive and/or social–emotional development in 2010 [3]. Thus, providing evidence on the association between housing and ECD may shed light upon a possibility to facilitate a faster attainment of the Sustainable Development Goals that advocate for ensuring universal achievement of appropriate ECD and access to adequate, safe, and affordable housing by 2030 [17].

In this study, we used Multiple Indicator Cluster Survey (MICS) data from 20 countries in SSA to test the hypothesis that improved housing is associated with a higher odds of being developmentally on track in either of the 2 ECD domains (i.e., cognitive or social–emotional) among children aged 36 to 59 months. Furthermore, we assessed the heterogeneity of the associations between subpopulations, based on the child’s gender, maternal education, and household wealth, which might influence ECD and/or housing quality [3,18]. To the best of our knowledge, this is the first comprehensive study examining the relationship between housing and ECD in SSA to date.

## Methods

This study is reported as per the Strengthening the Reporting of Observational Studies in Epidemiology (STROBE) guideline (S1 Checklist).

### Protocol

The prospective analysis plan is included as S1 Analysis Plan. During the peer review process, we made several changes to the analysis plan as per the reviewers’ suggestions: (1) changes in the outcomes, including the removal of the literacy–numeracy domain of the ECDI and replacement of the term “delay” with “on-track” to describe the outcomes; (2) changes in the a priori confounding variables, including adding the availability of books and playthings and treating the age of the child and the child’s household wealth index as linear variables instead of dichotomizing them; and (3) changes in the statistical analysis by applying the conditional logistic model instead of the standard logistic model. This list of changes is also included at the end of S1 Analysis Plan.

### Data source

MICS are nationally representative cross-sectional household surveys carried out in LMIC every 5 years [19]. MICS data of SSA countries were included in the analysis if (1) data were from the most recent round of MICS; (2) information on housing quality and ECD was included; and (3) data were publicly available on the MICS website (http://mics.unicef.org/surveys) before May 2020. Registration and login details were required to access the data.

MICS data are internationally comparable due to the use of standardized and validated questionnaires and a uniform 2-stage sampling design. The first stage was cluster formation through the probability proportional to size sampling, based on the most recent national census. In the second stage, the random sampling method was used to select a predetermined number (20 to 30) of households from the clusters. Standardized face-to-face interviews were conducted to obtain information from the household members [19].

### Outcomes

ECD was measured by the Early Childhood Development Index (ECDI), which is a questionnaire introduced to the MICS since 2010. The ECDI provides the largest reliable ECD data of children aged 36 to 59 months in LMIC [20]. The 10-item ECDI was developed through pilot tests of the 18-item ECDI in the Philippines, Kenya, and Jordan, which was validated with a Cronbach’s alpha of 0.7 or above on all 4 domains (cognitive, approaches to learning, emotional, and language) [20], as well as through revisions by child development experts [20]. According to the confirmatory factor analysis conducted with children aged 36 to 59 months in 35 LMIC, ECDI demonstrated adequate model fit for the original domains proposed by ECDI developers [3].

The ECDI has 10 items, divided into 4 developmental domains: physical, learning/cognition, social–emotional, and literacy–numeracy [20]. S1 Table presents the complete ECDI questionnaire. Children were classified as developmentally on-track in a certain domain if they failed no more than 1 ECDI item in that domain [20].

To be consistent with previous studies evaluating the global ECD status and exploring the factors that affect ECD [3,21,22], we only selected the development in the cognitive and social–emotional domains as outcomes. Skills covered by these domains are core developmental milestones for children aged 36 to 59 months, such as following commands, independence, and concentration [23].

The physical and literacy–numeracy domains were excluded from this study for several reasons. The pre-academic knowledge, assessed in the literacy–numeracy domain, is substantially more advanced than the literacy–numeracy skills assessed in other questionnaires for this age range (e.g., the Ages & Stages Questionnaire) [24,25]. Therefore, those items are more likely to reflect social norms around early education than children’s developmental capacity [3]. The development of pincer grasp, assessed in the physical domain, was usually achieved before 12 months of age [26]. Consequently, the sensitivity of this item was low [3], resulting in an extremely large number of children being developmentally on track in the physical domain, which was difficult for conducting statistical modeling [27].

### Exposures

We defined improved housing based on the definition developed by the United Nations [28,29]. Accordingly, an improved housing has all of the 4 following characteristics: (1) Access to improved water source; (2) Access to improved sanitation facilities; (3) Sufficient living area; and (4) Housing durability. The fifth characteristic “Security of tenure” proposed by the United Nations was excluded from our definition because of the lack of internationally comparable data [16]. Our definition was aligned with 2 recent studies that explored changes in housing and their impact on child health in SSA [9,16].

Table A in S1 Text shows the measurement criteria for the improved water source and sanitation facilities, as outlined by the World Health Organization and United Nations Children’s Fund Joint Monitoring Programme for Water Supply, Sanitation and Hygiene [30]. Living area was considered sufficient for household members if no more than 3 people shared the same habitable room [31]. A house was considered to have a housing durability if at least 2 out of 3 of the materials for the walls, roof, and floor were finished [9,16]. Table B in S1 Text presents the MICS classifications of finished and unfinished materials.

### A priori confounding variables

To reduce confounding bias, we included the following covariates: age (months) and gender of the child, maternal education, household wealth index, and the availability of children’s books and playthings. The age of the child was included as a continuous variable. Maternal education was included as a categorical variable, with different classifications across the countries. Most of the countries categorized women into “no education,” “primary education,” and “secondary or higher education” based on the highest education level they achieved. In Lesotho, women with no education were combined with those who had only basic primary education. In Ghana, maternal education was classified into none, primary, middle, and secondary or higher. In Mauritania, the categories were none, Koranic, primary, and secondary or higher. In Nigeria, the categories were none, nonformal, primary, and secondary or higher.

The construction of the wealth index in MICS includes selecting a basket of asset indicator variables, running a principal component analysis, and calculating wealth scores that are based on the first component of the principal component analysis and assigning the score to household members [32]. However, the standard asset indicator variables usually include housing materials, water, and sanitation [32]. A bespoke wealth index excluding these exposure variables is recommended by MICS to avoid concerns relating to tautology, which would bias the estimate toward null [33]. Aligned with previous studies [9,16], we selected the ownerships of the following durable assets as the asset indicator variables: (1) car; (2) motorboat; (3) scooter; (4) cart; (5) bicycle; (6) television; (7) refrigerator; (8) radio; (9) watch; (10) mobile telephone; (11) landline telephone; and (12) electrification of the household. These variables were commonly used in MICS to construct the wealth index [32]. In each country, we excluded indicators where <5% or >95% of households owned the asset. The tailored wealth score was included as a continuous variable as a covariate [9]. When we conducted subgroup analyses according to household wealth, the wealth score was not included in the model as a covariate. The total household population for each country was divided into quintiles based on their tailored wealth score, arranged from poorest (wealth quintile 1) to richest (wealth quintile 5). Children were divided into those belonging to the lower 2 quintiles and those belonging to the upper 3 quintiles according to the wealth quintile of the household they were living in.

We controlled for the availability of children’s books and playthings to account for the variability in individual learning resources [18,34]. The availability of children’s books was defined as having 3 or more children’s books at home in the MICS indicator list [34]. However, the median number of books at home was 0 in all the 20 countries. Therefore, we set the threshold for the availability of children’s books as the presence of at least 1 children’s book or picture book at home, which was consistent with the previous literature [35]. The availability of playthings was defined as the availability of at least 2 types of the following 3 types of playthings at home: (1) homemade toys; (2) toys from a shop or manufactured toys; and (3) household objects or objects found outside [34].

### Statistical analysis

In each country, we applied a conditional logistic model to investigate the relationships between housing and ECD. In conditional logistic regression, children living in different housing environments were only compared within the same cluster. Therefore, this approach minimized neighborhood-level confounding factors, such as availability of education services, ECD programs, social norms, and other environmental determinants [8,9].

The outcome of interest was the on-track development in each of the 2 ECD domains, and the exposures were one of the 4 improved housing characteristics, and improved housing as a combined variable. First, we ran a set of models for each individual characteristic of improved housing on the ECD outcomes. The combined variable (improved housing) considering the 4 characteristics was analyzed in a similar manner. Then, the 4 characteristics of improved housing were explored simultaneously in the same model to quantify the extent of the contribution made by each characteristic. In all models, we controlled the same set of a priori confounding variables.

The overall rate of missing data in most countries was below 5%, except for Sierra Leone (5.0%), Mauritania (9.9%), and Chad (10.7%). Missingness was not >10% for any variable in any country (S2 Table). With the assumption of missing completely at random, we used a complete case approach to analyze the data, which is consistent with previous research [9]. Therefore, only children without any missing information on the ECDI, confounding variables, and all the exposures were included in the model.

We assessed the heterogeneity of the estimated associations among countries using the I^2^ index [36]. We then conducted random-effects meta-analyses with the DerSimonian and Laird method [37] to relate the country-specific odds ratio (OR) of improved housing on ECD to the pooled estimates for the 20 SSA countries.

To further reveal the heterogeneity of the association between improved housing and the odds of being developmentally on track in each ECD domain across different populations, we examined the factors that were expected to impact ECD and/or housing [3,18], including the child’s gender, maternal education (having completed less than secondary education versus secondary or higher education), and household wealth (belonging to the lower 2 versus upper 3 wealth quintiles). We incorporated the 2-way interaction term between improved housing and an additional factor in each country. We computed the expected OR for the association between improved housing and on-track development in each ECD domain for each level of the factor. The overall OR for each subgroup was calculated using a random-effects model. In each country, the interaction was assessed on a multiplicative scale by calculating the OR of the interaction term between improved housing and the factor [38], which was then pooled for all countries using a random-effects model.

A *p*-value of 0.05 was considered as significant, with all analyses being conducted in SPSS version 25.0 and R version 3.6.1.

### Ethics statement

No human participant work or field research was conducted as part of this secondary data analysis. All data used are publicly available and fully de-identified. Ethical approval for the MICS was provided by individual review boards within each participating country at the time of survey implementation.

## Results

### Study population

Data were extracted from surveys in 20 countries dating from 2010 to 2019. From the total population of 92,433 children aged 36 to 59 months surveyed, complete outcomes, covariates, and exposures data were available for 88,271 children (Table 1). Across all surveys, the children’s average age was 47.2 months, and 49.8% were girls (Table 2). The proportion of rural residence ranged from 49.8% in Benin to 88.9% in Malawi. Zimbabwe, Congo, and Eswatini were the only 3 countries where the proportion of mothers with less than secondary education was lower than 50%. In Chad, 94.4% of the children’s mothers did not complete secondary education. Children’s books were less accessible than having at least 2 types of playthings. The proportion of children having no book at home ranged from 74.3% in Nigeria to 98.0% in DR Congo. The proportion of children having less than 2 types of playthings at home ranged from 21.1% in Zimbabwe to 62.9% in Guinea-Bissau.

**Table 1:** Survey characteristics of the 20 countries included in the analysis.

Country	MICS round	Year of investigation	Number of children aged 36–59 months (*N*)	Number of children aged 36–59 months without missing information (*N*)^a^
**Benin**	MICS5	2014	4,880	4,709
**Côte d’Ivoire**	MICS5	2016	3,730	3,598
**Cameroon**	MICS5	2014	2,846	2,739
**Chad**	MICS4	2010	7,139	6,375
**Congo**	MICS5	2015	3,675	3,526
**DR Congo**	MICS6	2018	8,704	8,453
**Eswatini**	MICS5	2014	1,091	1,038
**Gambia**	MICS6	2018	4,304	4,115
**Ghana**	MICS6	2018	3,668	3,521
**Guinea**	MICS5	2016	3,164	3,099
**Guinea-Bissau**	MICS5	2014	2,970	2,849
**Lesotho**	MICS6	2018	1,326	1,292
**Madagascar**	MICS6	2018	5,142	5,004
**Malawi**	MICS5	2014	7,839	7,536
**Mali**	MICS5	2015	6,550	6,318
**Mauritania**	MICS5	2015	4,446	4,005
**Nigeria**	MICS5	2013	11,648	11,101
**Sierra Leone**	MICS6	2014	4,810	4,568
**Togo**	MICS6	2011	1,989	1,946
**Zimbabwe**	MICS6	2019	2,512	2,479
**Total**	-	-	92,433	88,271

^a^Number of children without missing information on ECDI, confounding variables, and exposures.

ECDI, Early Childhood Development Index; MICS, Multiple Indicator Cluster Survey.

**Table 2:** Demographic characteristics of study participants.

Country	Number of children aged 36–59 months without missing information (*N*)^a^	Mean age (months)	Girls (%)	Rural residence (%)	Mother completed less than secondary education (%)	Having no book at home (%)	Having less than 2 types of playthings at home (%)
**Benin**	4,709	47.4	50.5	49.8	86.5	89.8	43.1
**Côte d’Ivoire**	3,598	46.8	48.9	74.0	90.4	93.8	47.6
**Cameroon**	2,739	46.8	50.7	55.1	67.4	82.1	34.7
**Chad**	6,375	48.0	50.5	62.7	94.4	96.6	40.0
**Congo**	3,526	48.2	48.7	69.6	46.0	89.8	43.8
**DR Congo**	8,453	46.7	51.4	73.3	64.7	98.0	58.1
**Eswatini**	1,038	47.4	48.7	85.4	47.8	82.6	22.7
**Gambia**	4,115	47.5	48.5	63.4	79.0	94.6	43.0
**Ghana**	3,521	47.2	50.9	60.2	87.1	76.0	43.5
**Guinea**	3,099	47.3	48.4	70.3	88.3	95.5	62.2
**Guinea-Bissau**	2,849	47.0	50.4	72.9	89.4	97.3	62.9
**Lesotho**	1,292	47.8	50.5	75.5	58.1	89.9	34.3
**Madagascar**	5,004	47.3	49.5	77.2	79.1	94.6	40.5
**Malawi**	7,536	47.3	50.2	88.9	85.1	95.3	46.0
**Mali**	6,318	46.3	48.5	78.3	90.0	96.4	34.6
**Mauritania**	4,005	46.8	50.6	57.1	86.4	91.7	56.6
**Nigeria**	11,101	47.2	49.2	73.5	65.4	74.3	44.5
**Sierra Leone**	4,568	47.3	49.7	71.3	80.4	91.7	50.7
**Togo**	1,946	47.2	46.7	69.9	75.0	94.1	54.9
**Zimbabwe**	2,479	47.6	50.0	70.3	38.1	86.0	21.1
**Total**	88,271	47.2	49.8	70.6	77.0	90.5	45.5

^a^Number of children without missing information on ECDI, confounding variables, and exposures.

ECDI, Early Childhood Development Index.

### Improved housing

Across the 20 countries, the median prevalence of improved housing was 18.4%, ranging from 39.8% in Zimbabwe to 3.6% in Madagascar. The median prevalence of finished building materials, improved drinking water, improved sanitation facilities, and sufficient living area were 61.5%, 70.5%, 43.8%, and 63.2%, respectively (Table 3). Across all surveys, the prevalence of improved housing was not statistically different between boys versus girls (17.2% versus 16.7%, *p* = 0.067), but significantly different between children whose mother completed less than secondary versus secondary or higher education (11.4% versus 35.5%, *p* < 0.001) and children with household wealth belonging to the lower 2 versus upper 3 quintiles (5.8% versus 26.6%, *p* < 0.001).

**Table 3:** Characteristics of housing qualities of study participants.

Country	Number of children aged 36–59 months without missing information (*N*)^a^	Finished building materials (%)	Improved drinking water (%)	Improved sanitation facilities (%)	Sufficient living area (%)	Improved housing (%)
**Benin**	4,709	69.6	73.5	34.8	56.9	17.4
**Côte d’Ivoire**	3,598	73.7	76.8	36.2	61.1	18.2
**Cameroon**	2,739	59.0	69.7	49.7	70.6	28.3
**Chad**	6,375	16.7	54.8	16.1	48.5	4.3
**Congo**	3,526	59.8	67.4	29.8	60.7	17.4
**DR Congo**	8,453	19.4	36.9	27.0	59.9	4.5
**Eswatini**	1,038	88.1	67.3	76.5	66.9	38.0
**Gambia**	4,115	89.6	88.1	48.4	77.8	33.7
**Ghana**	3,521	85.1	83.9	52.5	53.5	23.6
**Guinea**	3,099	71.1	78.5	48.0	65.7	24.5
**Guinea-Bissau**	2,849	28.5	68.2	11.9	74.3	5.6
**Lesotho**	1,292	63.2	85.8	65.3	51.5	28.3
**Madagascar**	5,004	23.2	31.5	8.9	29.8	3.6
**Malawi**	7,536	34.8	85.1	59.8	69.7	17.4
**Mali**	6,318	39.2	66.3	41.8	65.4	15.7
**Mauritania**	4,005	45.0	80.1	45.8	35.2	18.6
**Nigeria**	11,101	63.7	65.5	46.7	54.3	19.4
**Sierra Leone**	4,568	50.4	57.1	37.8	73.1	16.6
**Togo**	1,946	84.0	71.3	36.9	65.9	20.6
**Zimbabwe**	2,479	79.4	75.2	64.1	71.3	39.8

^a^Number of children without missing information on ECDI, confounding variables, and exposures.

ECDI, Early Childhood Development Index.

### Early childhood development

The median prevalence of on-track development in cognitive and social–emotional domains were 83.9% (ranging from 50.0% in Chad to 95.7% in Lesotho) and 71.4% (ranging from 61.4% in Congo to 79.3% in Lesotho), respectively (Table 4). The prevalence of on-track cognitive development was higher in girls (79.8% versus 79.2%, *p* = 0.034), in children whose mother completed secondary or higher education (85.3% versus 77.8%, *p* < 0.001), and in children with household wealth belonging to the upper 3 quintiles (82.0% versus 76.6%, *p* < 0.001). The prevalence of on-track social–emotional development was higher in girls (74.2% versus 68.4%, *p* < 0.001), in children whose mother completed secondary or higher education (72.6% versus 70.9%, *p* < 0.001), and in children with household wealth belonging to the upper 3 quintiles (71.8% versus 70.6%, *p* < 0.001).

**Table 4:** Status of ECD of study participants.

Country	Number of children aged 36–59 months without missing information (*N*)^a^	On-track cognitive development (%)	On-track social–emotional development (%)
**Benin**	4,709	83.0	73.7
**Côte d’Ivoire**	3,598	86.9	69.8
**Cameroon**	2,739	85.8	68.0
**Chad**	6,375	50.0	69.4
**Congo**	3,526	85.5	61.4
**DR Congo**	8,453	64.2	78.0
**Eswatini**	1,038	95.1	66.0
**Gambia**	4,115	95.5	63.5
**Ghana**	3,521	82.2	66.9
**Guinea**	3,099	79.6	62.3
**Guinea-Bissau**	2,849	86.3	75.9
**Lesotho**	1,292	95.7	79.3
**Madagascar**	5,004	84.3	76.1
**Malawi**	7,536	80.9	75.2
**Mali**	6,318	84.1	73.6
**Mauritania**	4,005	83.6	67.0
**Nigeria**	11,101	80.4	73.5
**Sierra Leone**	4,568	79.5	61.7
**Togo**	1,946	72.5	73.0
**Zimbabwe**	2,479	91.2	77.4

^a^Number of children without missing information on ECDI, confounding variables, and exposures.

ECD, early childhood development; ECDI, Early Childhood Development Index.

### Association between improved housing and ECD

**Fig 1:**
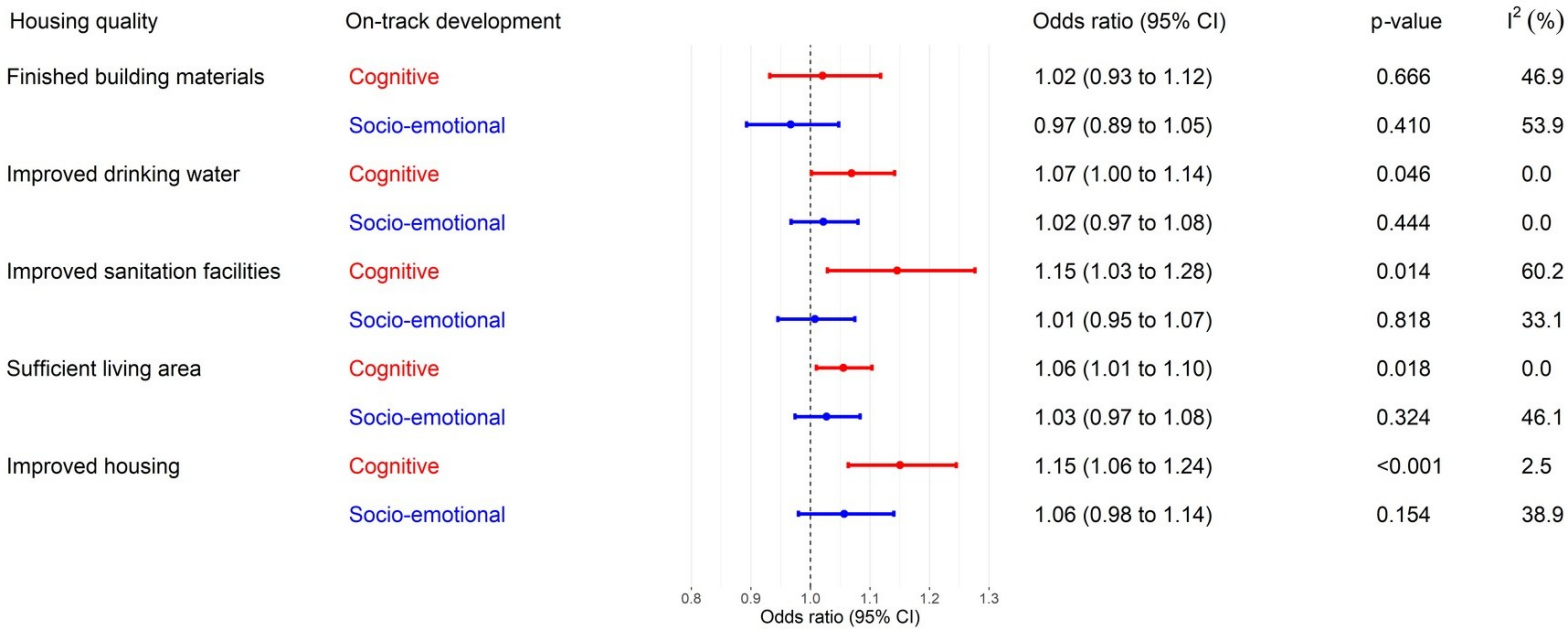
Association between housing quality and ECD in children aged 36–59 months in SSA. Odds ratios are adjusted for age (months) and gender of the child, maternal education, household wealth index, and the availability of children’s books and playthings. CI, confidence interval; ECD, early childhood development; SSA, sub-Saharan Africa.

Fig 1 shows the associations between improved housing and ECD as estimated by the meta-analyses, adjusted for a priori confounding variables. Improved housing was associated with a 15% increase in the odds of being developmentally on track in the cognitive domain (OR = 1.15, 95% CI 1.06 to 1.24, *p* < 0.001). Similar associations were found for improved drinking water (OR = 1.07, 95% CI 1.00 to 1.14, *p* = 0.046), improved sanitation facilities (OR = 1.15, 95% CI 1.03 to 1.28, *p* = 0.014), and sufficient living area (OR = 1.06, 95% CI 1.01 to 1.10, *p* = 0.018). Finished building materials was not associated with on-track cognitive development (OR = 1.02, 95% CI 0.93 to 1.12, *p* = 0.666). Overall, there was no association between improved housing and on-track social–emotional development (OR = 1.06, 95% CI 0.98 to 1.14, *p* = 0.154). The unadjusted associations between improved housing and ECD are shown in S1 Fig. We detected small to moderate heterogeneity between countries for most of the associations, except for the association between improved sanitation facilities and on-track cognitive development (I^2^ = 60.2), which is positive and statistically significant in Chad, DR Congo, Gambia, Guinea, Guinea-Bissau, and Nigeria (Figure E in S2 Text).

S2 Fig shows the associations in the models where we simultaneously adjusted for all 4 improved housing characteristics and a priori confounding variables. Improved sanitation facilities (OR = 1.14, 95% CI 1.02 to 1.27, *p* = 0.020) and sufficient living area (OR = 1.05, 95% CI 1.01 to 1.10, *p* = 0.025) were independently associated with a higher likelihood of being developmentally on track in the cognitive domain, while the association between improved drinking water and on-track cognitive development was no longer statistically significant (OR = 1.06, 95% CI 0.99 to 1.13, *p* = 0.094).

**Fig 2:**
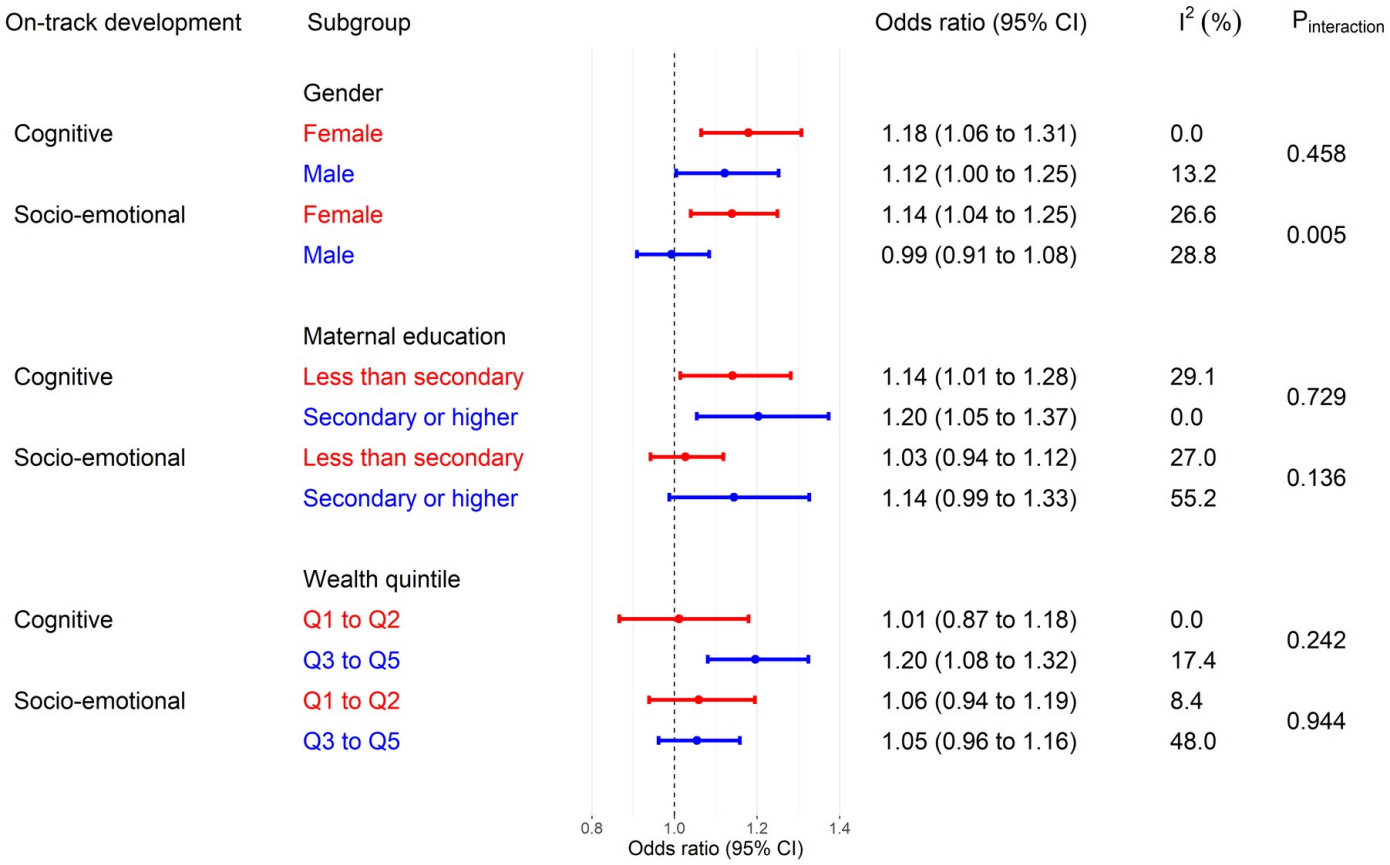
Subgroup analyses of the association between improved housing and ECD in children aged 36–59 months in SSA. Odds ratios are adjusted for age (months) and gender of the child, maternal education, household wealth index, and the availability of children’s books and playthings. P_interaction_, *p*-value for the odds ratio of the interaction term between improved housing and the factor (i.e., *p*-value for multiplicative interaction); Q1 to Q2, wealth quintile 1 to wealth quintile 2; Q3 to Q5, wealth quintile 3 to wealth quintile 5. ECD, early childhood development; SSA, sub-Saharan Africa.

Fig 2 shows the subgroups analyses of the associations between improved housing and ECD by the child’s gender, maternal education, and household wealth quintiles. The association between improved housing and on-track social–emotional development was stronger among girls (*p* for interaction = 0.005). Improved housing was associated with a 14% increase in the odds of being developmentally on track in the social–emotional domain in girls (OR = 1.14, 95% CI 1.04 to 1.25, *p* = 0.006). This association was not detected in boys (OR = 0.99, 95% CI 0.91 to 1.08, *p* = 0.875). The association between improved housing and on-track cognitive development was significant among children with levels of household wealth in the upper 3 quintiles (OR = 1.20, 95% CI 1.08 to 1.32, *p* < 0.001) but not among children with levels of household wealth in the lower 2 quintiles (OR = 1.01, 95% CI 0.87 to 1.18, *p* = 0.893), although the differences between these 2 groups were not statistically significant (*p* for interaction = 0.242).

## Discussion

To the best of our knowledge, this is the first multicountry study in SSA examining the association between improved housing and ECD. Overall, we found that improved housing was associated with 15% higher odds of being developmentally on track in the cognitive domain. Individually, we found that improved drinking water, improved sanitation facilities, and sufficient living area were associated with on-track cognitive development. When all housing characteristics were included in the same model, the results suggest that improved sanitation facilities and sufficient living area were independently associated with a higher likelihood of being developmentally on track in the cognitive domain. Across all children, none of the improved housing characteristics or improved housing as a combined variable were associated with on-track social–emotional development. However, we observed a significant association between improved housing and on-track social–emotional development among girls.

Our findings are consistent with prior studies conducted in LMIC that investigated the associations between water and sanitation on cognitive development. A study using MICS data on housing and the Ten Questions Scale to measure ECD suggested that children with more access to protected water or flush toilet had a lower likelihood of cognitive impairment in Thailand and Mongolia [39]. A meta-analysis of studies conducted in Indonesia, Brazil, Pakistan, and India suggested that access to household sanitation was associated with improved cognitive ability in children [40]. A prospective cohort study showed that flush toilet use in infancy was associated with higher cognitive scores in children aged between 18 and 36 months in Tanzania, whereas access to improved water in infancy was not associated with later cognitive development [41].

Previous studies have shown inconsistent associations between building materials and ECD. A study of low-income urban neighborhoods in 3 US cities reported a correlation between the poor physical quality of the house (e.g., leaking roofs, broken windows, and rodents) and lower average reading and math skills in children with an average age of 10 [12]. However, another study showed that earth or dung flooring was not associated with cognitive impairment in children aged between 3 and 9 years in Bangladesh, Lao PDR, Mongolia, and Thailand [39]. We found no association between building materials and ECD. It is possible that a very high standard of improvements, rather than only having finished materials, is needed to promote ECD in LMIC. The association between sufficient living area and ECD has been documented mainly in high-income countries. Residential density (measured as the ratio of the number of people in the household to the number of rooms) during early childhood predicts cognitive development at 36 months both concurrently and prospectively in the US and the United Kingdom [42]. We extended these associations to preschoolers in SSA by showing the positive associations between sufficient living area and on-track cognitive development.

Findings of randomized controlled trials have shown inconsistent associations of water and sanitation intervention with social–emotional development. A trial done in Bangladesh found significant benefits of upgrading latrines and improving water quality on communication and personal social skills at 2 years of age [43]. Similar interventions in Kenya and Zimbabwe showed no effects on children’s social–emotional development [44,45]. We found that on-track social–emotional development was not associated with any of the housing qualities. It is possible that other components of nurturing care in the Nurturing Care Framework [4], such as adequate nutrition and responsive caregiving, would exert an influence on social–emotional development above housing environment [22,45,46]. Moreover, social–emotional development might be more susceptible to neighborhood context than housing environment [47,48].

Several mechanisms might explain the relationship between housing quality and cognitive development. First, improved housing quality can be associated with on-track cognitive development by protecting a child’s physical health. Notably, enteric infections, including intestinal worms caused by poor sanitation, can lead to iron deficiency, inflammation, and poor growth and development [49]. Diarrhea in early childhood has been linked to negative cognitive outcomes [50–52], and the sanitation intervention may reduce children’s exposure to this factor [53]. Second, improved housing quality may also be linked with on-track cognitive development by protecting parental mental health [54,55], which could potentially affect parent–child interactions [56,57]. Water and sanitation interventions have shown effectiveness in reducing maternal depressive symptoms [43]. Studies in high-income countries have shown that linkages between higher residential density and children’s poorer cognitive development are largely mediated by diminished maternal responsiveness [42].

We observed that the association between improved housing and on-track social–emotional development was stronger among girls than boys. Previous studies conducted in the US have shown that girls were more sensitive to housing interventions. Girls, rather than boys, who moved to low-poverty neighborhoods experienced significant reductions in conduct disorder and psychological distress, as well as an increase in education achievement [58–60]. Moreover, we observed that the association between improved housing and on-track cognitive development was relatively strong in children from relatively rich households; in poorer households, the association between improved housing and ECD may be masked by the larger impact of other risk factors of ECD. For example, a study conducted in Southeast Asia showed that stunting was associated with 28% lower odds of being developmentally on track in ECDI cognitive domain [21]. A study including 17 countries in Europe, Asia, and Africa showed that having books (OR = 1.62) and caregiver counting with the child (OR = 1.47) were associated with on-track cognitive development [22].

This study had several limitations. First, the causal relationship between improved housing and ECD remains unclear, due to the cross-sectional survey design and the residual confounding, which cannot be completely ruled out. Although we controlled for basic proxies of socioeconomic status, especially household materials and learning resources, as well as contextual effects, these a priori factors might not be sufficient for accounting for all potential confounders. Thus, the association between housing and ECD might be distorted due to potential selection bias. Second, the MICS does not contain adequate comprehensive measures for our hypothesized mediators. Additionally, the survey design restricted the causal mediation analysis. Therefore, it is important for future studies to elucidate potential mechanisms in this relationship. Third, other aspects of housing quality may be associated with ECD such as lead paint, rodents, and poor ventilation [61]. However, these characteristics have not been assessed in the MICS. Fourth, although the ECDI has shown validity in field studies [20], this instrument is less sensitive than more complex cognitive assessments used with older age groups. Other aspects of cognition, such as pattern recognition and memory or cultural/context-specific developmental milestones, are not captured in the ECDI and may lead to an inaccurate evaluation of ECD. This inaccuracy would result in non-differential misclassification of the outcomes and bias the effect estimates downwards [62]. Fifth, our analysis only included SSA countries, therefore, the generalizability of our findings to other regions of the world should be done with caution. The heterogeneity of the associations was generally moderate, except for the association between improved sanitation and on-track cognitive development. We believe that random-effects (rather than fixed-effect) models would account for this heterogeneity. More research is needed in these countries to better understand the heterogeneity in these findings.

The main strength of this study is that we investigated the association between housing and ECD by including a multinational sample from 20 countries across SSA. The external validity of our findings was enhanced than the analyses restricted to 1 country. Furthermore, instead of conducting the 1-stage analysis based on the pooled data of all countries, we performed meta-analyses to combine the results of different countries, which varied the effect of confounders in each country and minimized bias [63]. Additionally, incorporating the national survey data in meta-analyses minimized the publication bias.

In SSA, previous research has predominantly focused on the effects of health, nutrition, and responsive caregiving on ECD. The WASH Benefits trial in Kenya assessed the independent and combined effects of water, sanitation, handwashing, and nutrition interventions on children’s social–emotional development [43–45]. However, for other aspects of housing quality, such as finished building materials, sufficient living area, and overall housing environment, the evidence base for interventions remains limited. Therefore, given the present intensity of evidence, our study did not imply that scarce resources should be shifted away from services that already have strong evidence of supporting ECD (e.g., preprimary programming and home visiting services) [5]. Instead, our study highlights the need for studies to compare the effect sizes of housing with that of other factors (e.g., nutrition and parenting) on ECD, which is crucial for selecting the target factors of the interventions.

In conclusion, to the best of our knowledge, this is the first study in SSA to demonstrate the associations between multiple aspects of housing quality and children’s on-track development in cognitive and social–emotional domains. Given the cross-sectional nature of the study and the less nuanced representations of ECD outcomes using the ECDI, this study is inherently exploratory/hypothesis generating. Future work to establish a causal link between housing and ECD and to assess its underlying mechanisms is crucial for designing cost-effective ECD interventions and screenings in SSA and thereby, for supporting children in attaining their developmental milestones.

## Supporting information

S1 Analysis Plan Prospective analysis plan. (DOCX)

S1 Table The ECDI questionnaire. ECDI, Early Childhood Development Index.
(DOCX)

S2 Table Number and percentage of participants with missing data for each variable of interest in each country. (DOCX)

S1 Text Classifications of improved/unimproved facility types and finished/unfinished materials. (DOCX)

S2 Text Association between housing quality and ECD in children aged 36–59 months in SSA. ECD, early childhood development; SSA, sub-Saharan Africa.
(DOCX)

S1 Fig Unadjusted associations between housing quality and ECD in children aged 36–59 months in SSA. ECD, early childhood development; SSA, sub-Saharan Africa.
(DOCX)

S2 Fig Mutually adjusted association between housing quality and ECD in children aged 36–59 months in SSA. ECD, early childhood development; SSA, sub-Saharan Africa.
(DOCX)

S1 Checklist STROBE checklist. STROBE, Strengthening the Reporting of Observational Studies in Epidemiology.
(DOC)

## Abbreviations

ECD: early childhood development
ECDI: Early Childhood Development Index
LMIC: low- and middle-income countries
MICS: Multiple Indicator Cluster Survey
OR: odds ratio
SSA: sub-Saharan Africa
STROBE: Strengthening the Reporting of Observational Studies in Epidemiology
