# Supplementary material for: Housing environment and early childhood development in sub-Saharan Africa: A cross-sectional analysis
Source: PLoS Med. 2021 Apr 19;18(4):e1003578. doi: 10.1371/journal.pmed.1003578 (PMC8092764; doi:10.1371/journal.pmed.1003578)
Supplement: S1 Analysis Plan — (DOCX) [file pmed.1003578.s001.docx]

**S1 Analysis plan.** Prospective analysis plan.

During the revisions, we have made several changes to the analysis plan following the editors’ and reviewers’ suggestions. This prospective analysis plan is the original version. Details of the changes can be found at the end of this plan.

**Objective**

To test the hypothesis that unimproved housing is associated with higher odds of developmental delays in three early childhood development (ECD) domains (learning-cognition, literacy-numeracy, and socio-emotional development) among children aged 36 to 59 months, and if the association varies by gender, residence of the child, and the country’s income group.

**Data source**

Multiple Indicator Cluster Surveys (MICS) are population-based cross-sectional household surveys carried out at approximately 5-year intervals in a range of countries, mainly LMICs [1]. We will extract data from the most recent round of MICS completed in SSA countries that: (1) included information on housing quality and ECD; (2) were nationally (rather than subnational) representative; and (3) were publicly available on the MICS website (http://mics.unicef.org) before May 2020.

**Outcome**

The MICS contained Early Childhood Development Index (ECDI) data that was collected as a questionnaire. The 10-item ECDI assesses four domains of development: literacy-numeracy, physical, social-emotional, and learning [2]. We will define children’s developmental delay in each domain as the child failing more than one item in the domain [2]. We will select the developmental delay in the literacy-numeracy, learning, and social-emotional domains as outcomes. The physical domain will be excluded from this study because the sensitivity of was low [3,4].

**Exposure**

We will define unimproved housing based on the definition of slums developed for the Sustainable Development Goals by the United Nations [5]. We will classify an unimproved house as one in which the inhabitants suffer one or more of the following household deprivations: (1) Lack of access to improved water source; (2) Lack of access to improved sanitation facilities; (3) Lack of sufficient living area; and (4) Lack of housing durability. The fifth dimension of deprivation “Lack of security of tenure” proposed by the UN will be excluded from our definition for the lack of internationally comparable data [5]. The measurement criteria for the unimproved water source and sanitation facilities will be aligned with The World Health Organization and United Nations Children’s Fund (WHO/UNICEF) Joint Monitoring Programme for Water Supply, Sanitation and Hygiene [6]. Living area will be considered insufficient for the household members if more than three people share the same habitable room [5]. A house will be considered to have a lack of housing durability if more than one out of three of the materials for the walls, roof. and floor were unfinished [7,8].

***A priori* confounding variables**

We will include the following covariates: age (months) and gender of the child, maternal education, urban or rural residence, and household wealth. The construction of the wealth index in MICS includes selecting a basket of asset indicator variables, which usually include housing materials, water, and sanitation [9]. Aligned with previous studies [7,8], we will construct a bespoke wealth index excluding these exposure variables is recommended by MICS to avoid concerns relating to tautology. We will select the ownerships of the durable assets as the asset indicator variables, which were commonly used in MICS to construct the wealth index [9]. In each country, we will exclude indicators where <5% or >95% of households owned the asset.

**Statistical analysis**

In each country, we will investigate the relation between unimproved housing and ECD using multivariate logistic regressions. The outcome of interest will be the developmental delay in each of the three ECD domains and the exposure will be one of the four dimensions of household deprivations or unimproved housing. In all models, we will control the same set of *a priori* confounding variables. We will conduct random effects meta-analyses with the DerSimonian and Laird method [10] to relate the country-specific estimates for the effects of unimproved housing on ECD to the pooled estimates for the 20 SSA countries.

**References**

1. UNICEF. Multiple Indicator Cluster Survey (MICS) [cited May 22 2020]. Available from: http://mics.unicef.org.

2. UNICEF Data and Analytics Section. Development Of The Early Childhood Development Index In MICS Surveys: New York; 2017.

3. McCoy DC, Peet ED, Ezzati M, Danaei G, Black MM, Sudfeld CR, et al. Early Childhood Developmental Status in Low- and Middle-Income Countries: National, Regional, and Global Prevalence Estimates Using Predictive Modeling. PLOS medicine. 2016;13(6):e1002034.

4. Kang Y, Aguayo VM, Campbell RK, West KP. Association between stunting and early childhood development among children aged 36-59 months in South Asia. Matern Child Nutr. 2018;14 Suppl 4:e12684.

5. United Nations. SDG Indicators Metadata repository [cited May 23 2020]. Available from: https://unstats.un.org/sdgs/metadata/.

6. UNICEF, WHO. Progress on household drinking water, sanitation and hygiene 2000-2017: Special focus on inequalities. 2019.

7. Tusting LS, Bisanzio D, Alabaster G, Cameron E, Cibulskis R, Davies M, et al. Mapping changes in housing in sub-Saharan Africa from 2000 to 2015. Nature. 2019;568(7752):391-4.

8. Tusting LS, Gething PW, Gibson HS, Greenwood B, Knudsen J, Lindsay SW, et al. Housing and child health in sub-Saharan Africa: A cross-sectional analysis. PLOS Medicine. 2020;17(3):e1003055.

9. World Food Programme. Vulnerability Analysis & Mapping Guidance Paper-Creation of a Wealth Index. 2017.

10. DerSimonian R, Laird N. Meta-analysis in clinical trials. Control Clin Trials. 1986;7(3):177-88.

Please note that this prospective analysis plan is the original version. The improvements we made during the revisions include:

(1) We applied a conditional logistic model to minimize the confounding due to unmeasured neighborhood-level characteristics.

(2) We included the availability of books and playthings in the models to control the household learning resources.

(3) In order to maintain the variance that the covariates contribute to the model, in the first revision, we 1) included maternal education as categorical variable and adopted the country-specific classifications; and 2) included the age of the child and the child’s household wealth index as linear variables.

(4) The literacy-numeracy domain of the ECDI might be too advanced for children aged 36–59 months. Therefore, we removed this domain.

(5) The reviewers pointed out that the ECDI cannot measure developmental delays. Following the United Nations Children's Fund guidelines, we considered a child to be “developmentally on track” in a certain domain if the child failed no more than one item in that domain, and changed the reporting of the association between housing and ECD to be positive.
