# Supplementary material for: Housing environment and early childhood development in sub-Saharan Africa: A cross-sectional analysis
Source: PLoS Med. 2021 Apr 19;18(4):e1003578. doi: 10.1371/journal.pmed.1003578 (PMC8092764; doi:10.1371/journal.pmed.1003578)
Supplement: S1 Table — ECDI, Early Childhood Development Index. (DOCX) [file pmed.1003578.s002.docx]

**S1 Table.** The ECDI questionnaire.

| **Domain** | **Item** |
| --- | --- |
| **Literacy-numeracy** | (1) Can the child read at least four simple, popular words? (Yes/No) |
|  | (2) Does the child know the name and recognize the symbol of all numbers from one to ten? (Yes/No) |
|  | (3) Can the child identify or name at least ten letters of the alphabet? (Yes/No) |
| **Learning/cognition** | 1. Does the child follow simple directions on how to do something correctly? (Yes/No) |
|  | (2) When given something to do, is the child able to do it independently? (Yes/No) |
| **Social-emotional** | 1. Does the child get along well with other children? (Yes/No) |
|  | (2) Does the child kick, bite, or hit other children or adults? (Yes/No) (reverse coded) |
|  | (3) Does the child get distracted easily? (Yes/No) (reverse coded) |
| **Physical** | 1. Can the child pick up a small object with two fingers, like a stick or a rock from the ground? (Yes/No) |
|  | (2) Is the child sometimes too sick to play? (Yes/No) (reverse coded) |

ECDI: Early Childhood Development Index
