# Supplementary material for: Housing environment and early childhood development in sub-Saharan Africa: A cross-sectional analysis
Source: PLoS Med. 2021 Apr 19;18(4):e1003578. doi: 10.1371/journal.pmed.1003578 (PMC8092764; doi:10.1371/journal.pmed.1003578)
Supplement: S2 Table — (DOCX) [file pmed.1003578.s003.docx]

**S2 Table.** Number and percentage of participants with missing data for each variable of interest in each country.

| **Country** | **Number of children aged 36-59 months** | **Age** | **Gender** | **Residence** | **Wealth index** | **Maternal education** | |
| --- | --- | --- | --- | --- | --- | --- | --- |
|  | **N** | **N^a^** | **N^a^** | **N^a^** | **N^a^** | **N^a^** | **%^b^** |
| **Benin** | 4880 | 0 | 0 | 0 | 0 | 0 | 0.00 |
| **Côte d’Ivoire** | 3730 | 0 | 0 | 0 | 0 | 0 | 0.00 |
| **Cameroon** | 2846 | 0 | 0 | 0 | 0 | 2 | 0.07 |
| **Chad** | 7139 | 0 | 0 | 0 | 0 | 1 | 0.01 |
| **Congo** | 3675 | 0 | 0 | 0 | 0 | 1 | 0.03 |
| **DR Congo** | 8704 | 0 | 0 | 0 | 0 | 0 | 0.00 |
| **Eswatini** | 1091 | 0 | 0 | 0 | 0 | 3 | 0.27 |
| **Gambia** | 4304 | 0 | 0 | 0 | 0 | 6 | 0.14 |
| **Ghana** | 3668 | 0 | 0 | 0 | 0 | 0 | 0.00 |
| **Guinea** | 3164 | 0 | 0 | 0 | 0 | 1 | 0.03 |
| **Guinea-Bissau** | 2970 | 0 | 0 | 0 | 0 | 0 | 0.00 |
| **Lesotho** | 1326 | 0 | 0 | 0 | 0 | 0 | 0.00 |
| **Madagascar** | 5142 | 0 | 0 | 0 | 0 | 1 | 0.02 |
| **Malawi** | 7839 | 0 | 0 | 0 | 0 | 4 | 0.05 |
| **Mali** | 6550 | 0 | 0 | 0 | 0 | 0 | 0.00 |
| **Mauritania** | 4446 | 0 | 0 | 0 | 0 | 6 | 0.13 |
| **Nigeria** | 11648 | 0 | 0 | 0 | 0 | 2 | 0.02 |
| **Sierra Leone** | 4810 | 0 | 0 | 0 | 0 | 0 | 0.00 |
| **Togo** | 1989 | 0 | 0 | 0 | 0 | 0 | 0.00 |
| **Zimbabwe** | 2512 | 0 | 0 | 0 | 0 | 0 | 0.00 |
| **Total** | 92433 | 0 | 0 | 0 | 0 | 27 | 0.03 |

^a^ Number of participants with missing data for the variable of interest.

^b^ Percentage of participants with missing data for the variable of interest.

| **Country** | **Number of children aged 36-59 months** | **Book** | | **Playthings** | | **Building materials** | | **Drinking water** | |
| --- | --- | --- | --- | --- | --- | --- | --- | --- | --- |
|  | **N** | **N^a^** | **%^b^** | **N^a^** | **%^b^** | **N^a^** | **%^b^** | **N^a^** | **%^b^** |
| **Benin** | 4880 | 2 | 0.04 | 0 | 0.00 | 36 | 0.74 | 5 | 0.10 |
| **Côte d’Ivoire** | 3730 | 2 | 0.05 | 0 | 0.00 | 6 | 0.16 | 7 | 0.19 |
| **Cameroon** | 2846 | 0 | 0.00 | 5 | 0.18 | 23 | 0.81 | 5 | 0.18 |
| **Chad** | 7139 | 7 | 0.10 | 92 | 1.29 | 79 | 1.11 | 25 | 0.35 |
| **Congo** | 3675 | 1 | 0.03 | 3 | 0.08 | 6 | 0.16 | 7 | 0.19 |
| **DR Congo** | 8704 | 0 | 0.00 | 21 | 0.24 | 24 | 0.28 | 86 | 0.99 |
| **Eswatini** | 1091 | 0 | 0.00 | 1 | 0.09 | 0 | 0.00 | 0 | 0.00 |
| **Gambia** | 4304 | 0 | 0.00 | 4 | 0.09 | 1 | 0.02 | 1 | 0.02 |
| **Ghana** | 3668 | 0 | 0.00 | 0 | 0.00 | 10 | 0.27 | 3 | 0.08 |
| **Guinea** | 3164 | 5 | 0.16 | 6 | 0.19 | 24 | 0.76 | 0 | 0.00 |
| **Guinea-Bissau** | 2970 | 1 | 0.03 | 1 | 0.03 | 4 | 0.13 | 1 | 0.03 |
| **Lesotho** | 1326 | 0 | 0.00 | 2 | 0.15 | 1 | 0.08 | 6 | 0.45 |
| **Madagascar** | 5142 | 0 | 0.00 | 0 | 0.00 | 99 | 1.93 | 13 | 0.25 |
| **Malawi** | 7839 | 7 | 0.09 | 12 | 0.15 | 4 | 0.05 | 2 | 0.03 |
| **Mali** | 6550 | 3 | 0.05 | 5 | 0.08 | 9 | 0.14 | 8 | 0.12 |
| **Mauritania** | 4446 | 0 | 0.00 | 22 | 0.49 | 144 | 3.24 | 8 | 0.18 |
| **Nigeria** | 11648 | 24 | 0.21 | 30 | 0.26 | 80 | 0.69 | 36 | 0.31 |
| **Sierra Leone** | 4810 | 0 | 0.00 | 15 | 0.31 | 14 | 0.29 | 6 | 0.12 |
| **Togo** | 1989 | 0 | 0.00 | 3 | 0.15 | 9 | 0.45 | 4 | 0.20 |
| **Zimbabwe** | 2512 | 0 | 0.00 | 0 | 0.00 | 5 | 0.20 | 7 | 0.28 |
| **Total** | 92433 | 52 | 0.06 | 222 | 0.24 | 578 | 0.63 | 230 | 0.25 |

^a^ Number of participants with missing data for the variable of interest.

^b^ Percentage of participants with missing data for the variable of interest.

| **Country** | **Number of children aged 36-59 months** | **Sanitation facilities** | | **Living area** | | **Improved housing** | | **Cognitive development** | | **Social-emotional development** | |
| --- | --- | --- | --- | --- | --- | --- | --- | --- | --- | --- | --- |
|  | **N** | **N^a^** | **%^b^** | **N^a^** | **%^b^** | **N^a^** | **%^b^** | **N^a^** | **%^b^** | **N^a^** | **%^b^** |
| **Benin** | 4880 | 54 | 1.11 | 0 | 0.00 | 16 | 0.33 | 59 | 0.00 | 75 | 1.54 |
| **Côte d’Ivoire** | 3730 | 15 | 0.40 | 0 | 0.00 | 3 | 0.08 | 64 | 0.00 | 77 | 2.06 |
| **Cameroon** | 2846 | 3 | 0.11 | 0 | 0.00 | 4 | 0.14 | 50 | 0.18 | 61 | 2.14 |
| **Chad** | 7139 | 55 | 0.77 | 0 | 0.00 | 5 | 0.07 | 238 | 1.29 | 423 | 5.93 |
| **Congo** | 3675 | 20 | 0.54 | 0 | 0.00 | 10 | 0.27 | 74 | 0.08 | 89 | 2.42 |
| **DR Congo** | 8704 | 42 | 0.48 | 1 | 0.01 | 13 | 0.15 | 22 | 0.24 | 30 | 0.34 |
| **Eswatini** | 1091 | 2 | 0.18 | 0 | 0.00 | 0 | 0.00 | 18 | 0.09 | 25 | 2.29 |
| **Gambia** | 4304 | 6 | 0.14 | 0 | 0.00 | 2 | 0.05 | 90 | 0.09 | 104 | 2.42 |
| **Ghana** | 3668 | 17 | 0.46 | 0 | 0.00 | 6 | 0.16 | 9 | 0.00 | 30 | 0.82 |
| **Guinea** | 3164 | 0 | 0.00 | 0 | 0.00 | 2 | 0.06 | 22 | 0.19 | 20 | 0.63 |
| **Guinea-Bissau** | 2970 | 14 | 0.47 | 0 | 0.00 | 0 | 0.00 | 46 | 0.03 | 47 | 1.58 |
| **Lesotho** | 1326 | 3 | 0.23 | 0 | 0.00 | 2 | 0.15 | 2 | 0.15 | 7 | 0.53 |
| **Madagascar** | 5142 | 2 | 0.04 | 0 | 0.00 | 3 | 0.06 | 5 | 0.00 | 16 | 0.31 |
| **Malawi** | 7839 | 20 | 0.26 | 0 | 0.00 | 3 | 0.04 | 156 | 0.15 | 162 | 2.07 |
| **Mali** | 6550 | 9 | 0.14 | 0 | 0.00 | 7 | 0.11 | 97 | 0.08 | 151 | 2.31 |
| **Mauritania** | 4446 | 30 | 0.67 | 0 | 0.00 | 24 | 0.54 | 100 | 0.49 | 113 | 2.54 |
| **Nigeria** | 11648 | 24 | 0.21 | 0 | 0.00 | 22 | 0.19 | 161 | 0.26 | 189 | 1.62 |
| **Sierra Leone** | 4810 | 14 | 0.29 | 0 | 0.00 | 8 | 0.17 | 91 | 0.31 | 165 | 3.43 |
| **Togo** | 1989 | 8 | 0.40 | 0 | 0.00 | 5 | 0.25 | 6 | 0.15 | 9 | 0.45 |
| **Zimbabwe** | 2512 | 2 | 0.08 | 0 | 0.00 | 5 | 0.20 | 1 | 0.00 | 5 | 0.20 |
| **Total** | 92433 | 340 | 0.37 | 1 | 0.001 | 140 | 0.15 | 1311 | 1.42 | 1798 | 1.95 |

^a^ Number of participants with missing data for the variable of interest.

^b^ Percentage of participants with missing data for the variable of interest.
