# Supplementary material for: Housing environment and early childhood development in sub-Saharan Africa: A cross-sectional analysis
Source: PLoS Med. 2021 Apr 19;18(4):e1003578. doi: 10.1371/journal.pmed.1003578 (PMC8092764; doi:10.1371/journal.pmed.1003578)
Supplement: S1 Text — (DOCX) [file pmed.1003578.s004.docx]

**S1 Text.** Classifications of improved/unimproved facility types and finished/unfinished materials.

**Table A.** The World Health Organization and United Nations Children’s Fund Joint Monitoring Programme for Water Supply, Sanitation and Hygiene classifications of improved/unimproved facility types.

|  | **Drinking water** | **Sanitation** |
| --- | --- | --- |
| **Improved** | - Tap water in the dwelling, yard or plot - Public standposts - Boreholes / tubewells - Protected wells and springs - Rainwater - Packaged water, including bottled water and sachet water - Delivered water, including tanker trucks and small carts | - Flush and pour flush toilets connected to sewers - Flush and pour flush toilets or latrines connected to septic tanks or pits - Ventilated improved pit latrines - Pit latrines with slabs - Composting toilets, including twin pit latrines and container-based systems |
| **Unimproved** | - Unprotected wells and springs - Surface water | - Pit latrines without slabs - Hanging latrines - Bucket latrines - Open defecation |

**Table B.** Multiple Indicator Cluster Survey classifications of finished/unfinished materials.

|  | **Walls** | **Roof** | **Floor** |
| --- | --- | --- | --- |
| **Finished** | - Cement - Stone with lime / cement - Bricks - Cement blocks - Covered adobe - Wood planks / shingles - Burnt bricks - Metal - Bamboo with cement - Asbestos | - Metal - Wood - Calamine / Cement fibre - Ceramic tiles - Cement - Roofing shingles - Clay tiles - Asbestos | - Parquet or polished wood - Vinyl or asphalt strips - Ceramic tiles - Cement - Carpet - Terrazzo |
| **Unfinished** | - No Walls - Cane / Palm / Trunks - Dirt - Bamboo with mud - Stone with mud - Uncovered adobe - Plywood - Cardboard - Reused wood - Bamboo mat - Bamboo / bamboo with dry leaf - Bamboo lattice - Unburnt bricks - Straw - Mud blocks | - No Roof - Thatch / Palm leaf - Sod - Rustic mat - Palm / Bamboo - Wood planks - Cardboard - Canvas - Earth - Mud | - Earth / Sand - Dung - Wood planks - Palm / Bamboo - Stones - Matting |
