# Supplementary material for: Housing environment and early childhood development in sub-Saharan Africa: A cross-sectional analysis
Source: PLoS Med. 2021 Apr 19;18(4):e1003578. doi: 10.1371/journal.pmed.1003578 (PMC8092764; doi:10.1371/journal.pmed.1003578)
Supplement: S2 Text — ECD, early childhood development; SSA, sub-Saharan Africa. (DOCX) [file pmed.1003578.s005.docx]

**S2 Text.** Association between housing quality and early childhood development in children aged 36 to 59 months in sub-Saharan Africa (adjusted for age (months) and gender of the child, maternal education, household wealth index, and the availability of children’s books and playthings).


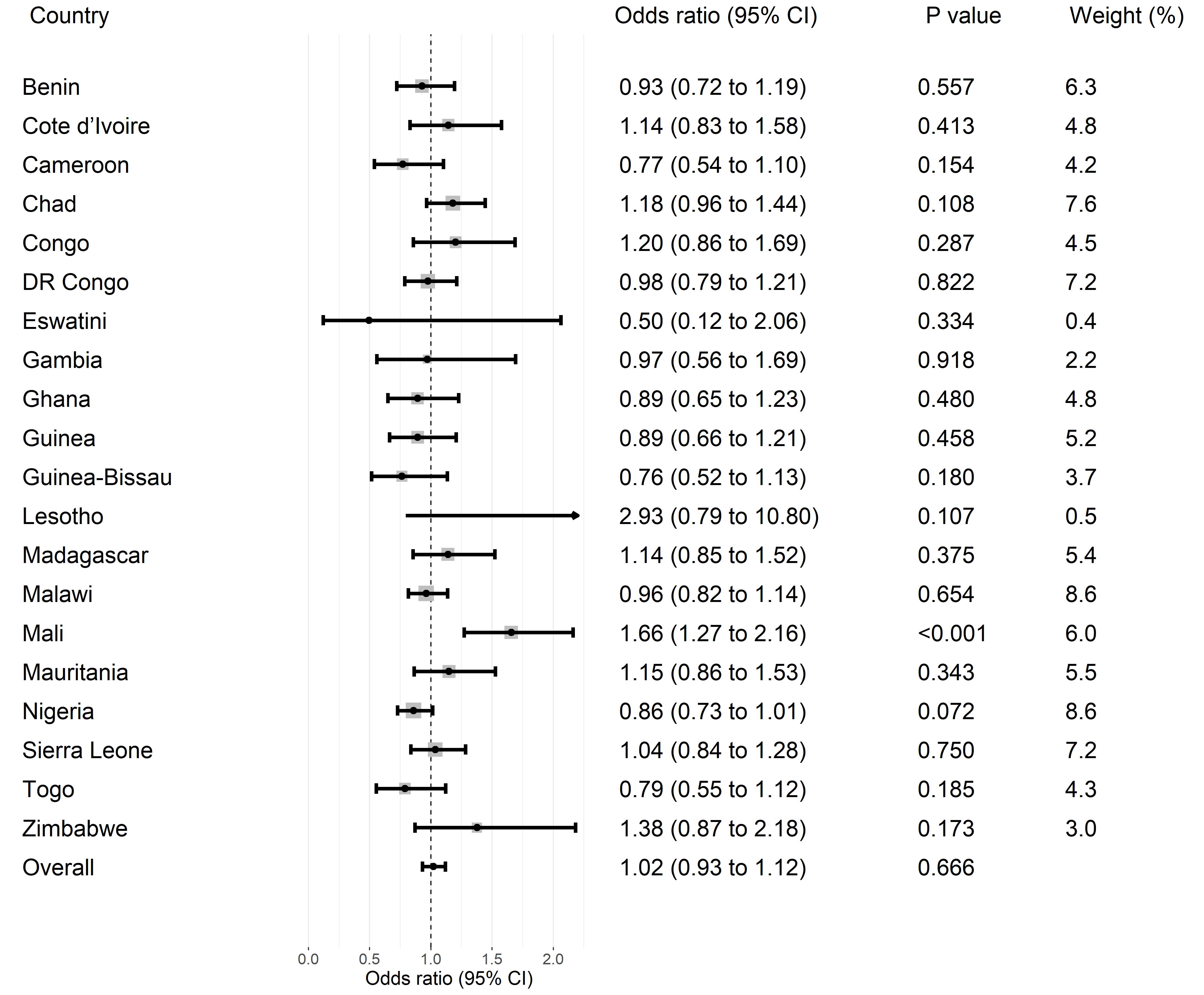
**Figure A.** Association between finished building materials and on-track cognitive development in children aged 36 to 59 months in sub-Saharan Africa.

CI: confidence interval; The weight given to each country is the inverse of the variance of the odds ratio estimate. Weight (%), which is indicated by the size of the box, is the proportion of the country’s weight that contributes to the sum of weights of all countries.


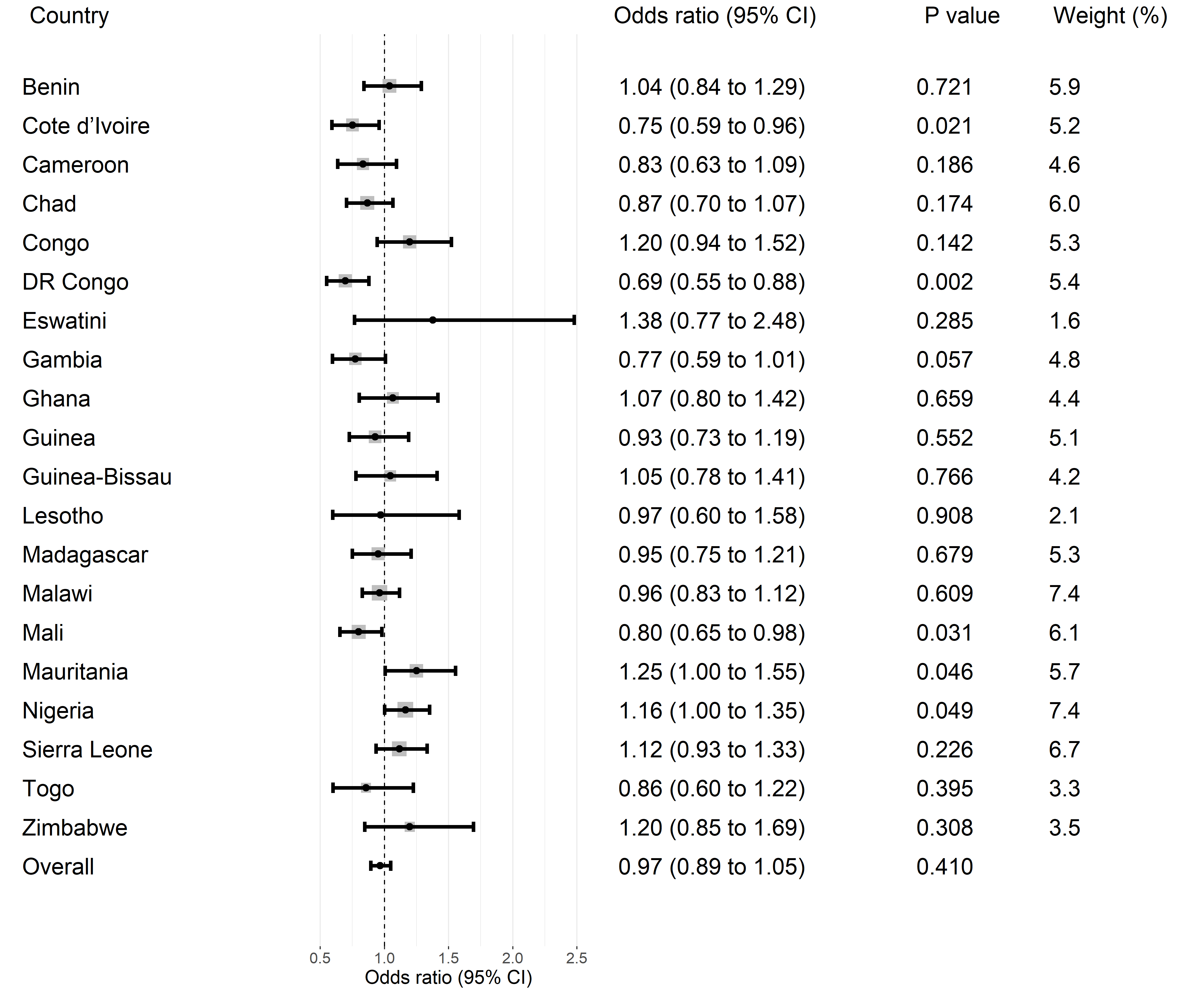
**Figure B.** Association between finished building materials and on-track social-emotional development in children aged 36 to 59 months in sub-Saharan Africa.

CI: confidence interval; The weight given to each country is the inverse of the variance of the odds ratio estimate. Weight (%), which is indicated by the size of the box, is the proportion of the country’s weight that contributes to the sum of weights of all countries.


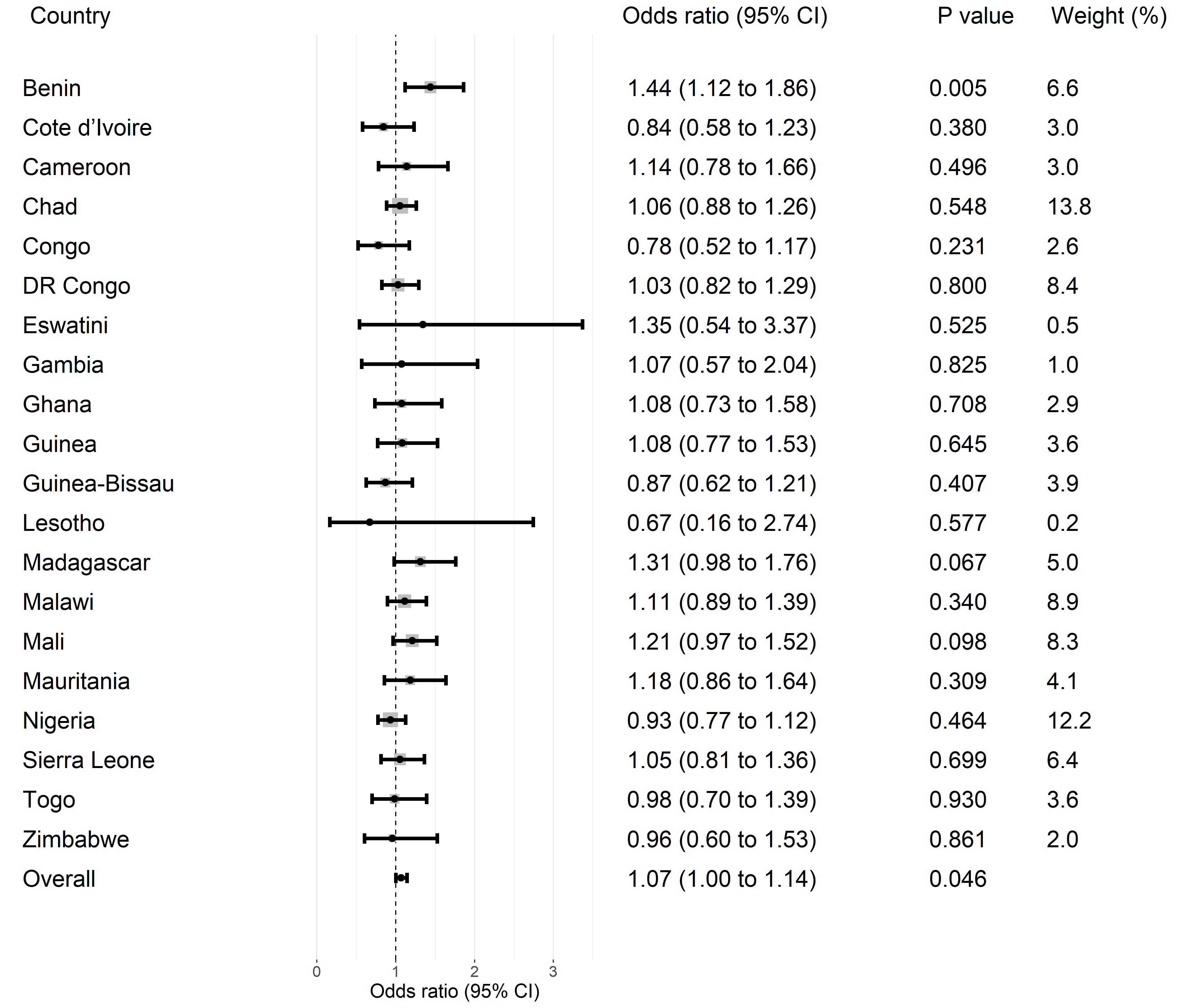
**Figure C.** Association between improved drinking water and on-track cognitive development in children aged 36 to 59 months in sub-Saharan Africa.

CI: confidence interval; The weight given to each country is the inverse of the variance of the odds ratio estimate. Weight (%), which is indicated by the size of the box, is the proportion of the country’s weight that contributes to the sum of weights of all countries.


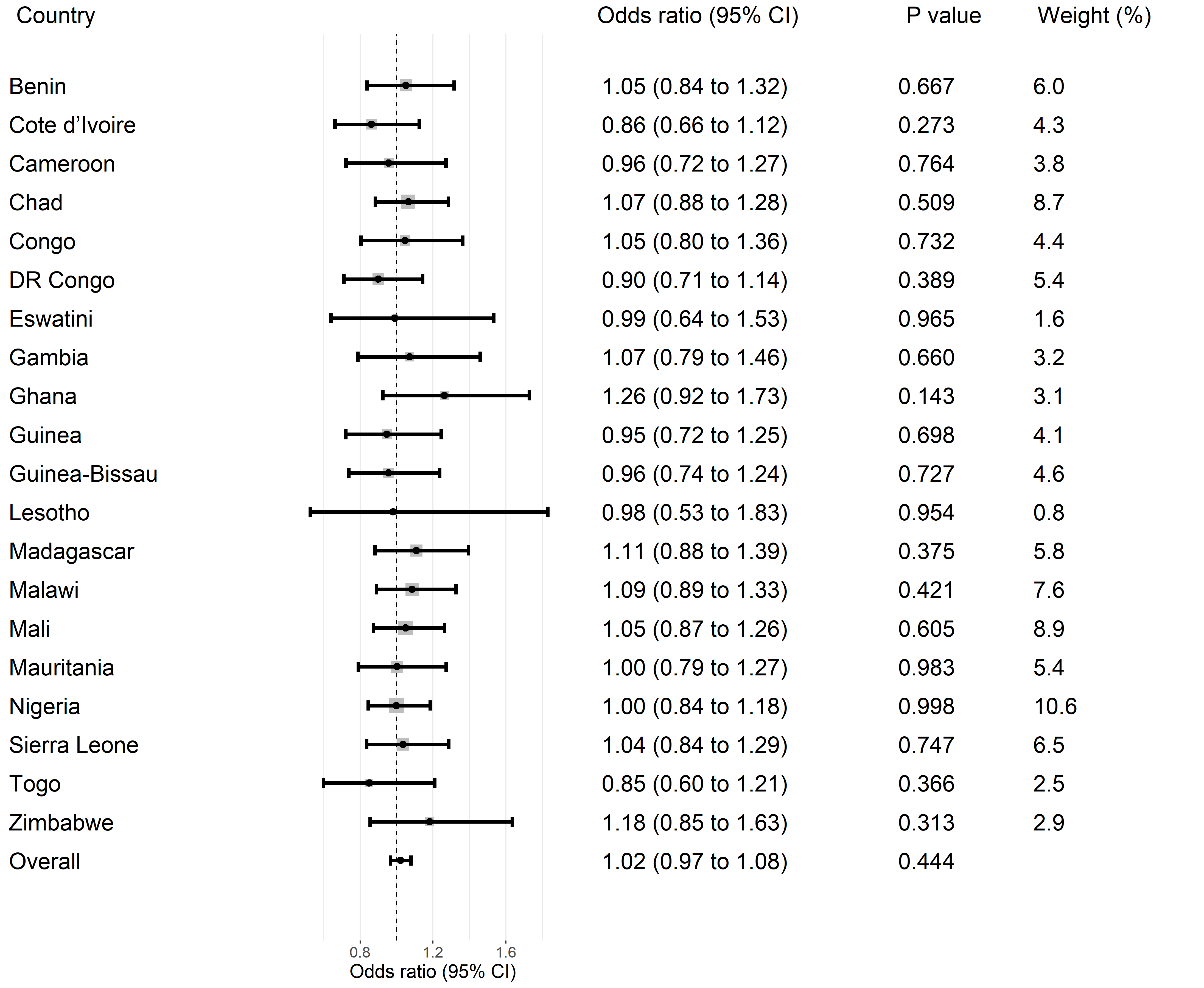
**Figure D.** Association between improved drinking water and on-track social-emotional development in children aged 36 to 59 months in sub-Saharan Africa.

CI: confidence interval; The weight given to each country is the inverse of the variance of the odds ratio estimate. Weight (%), which is indicated by the size of the box, is the proportion of the country’s weight that contributes to the sum of weights of all countries.


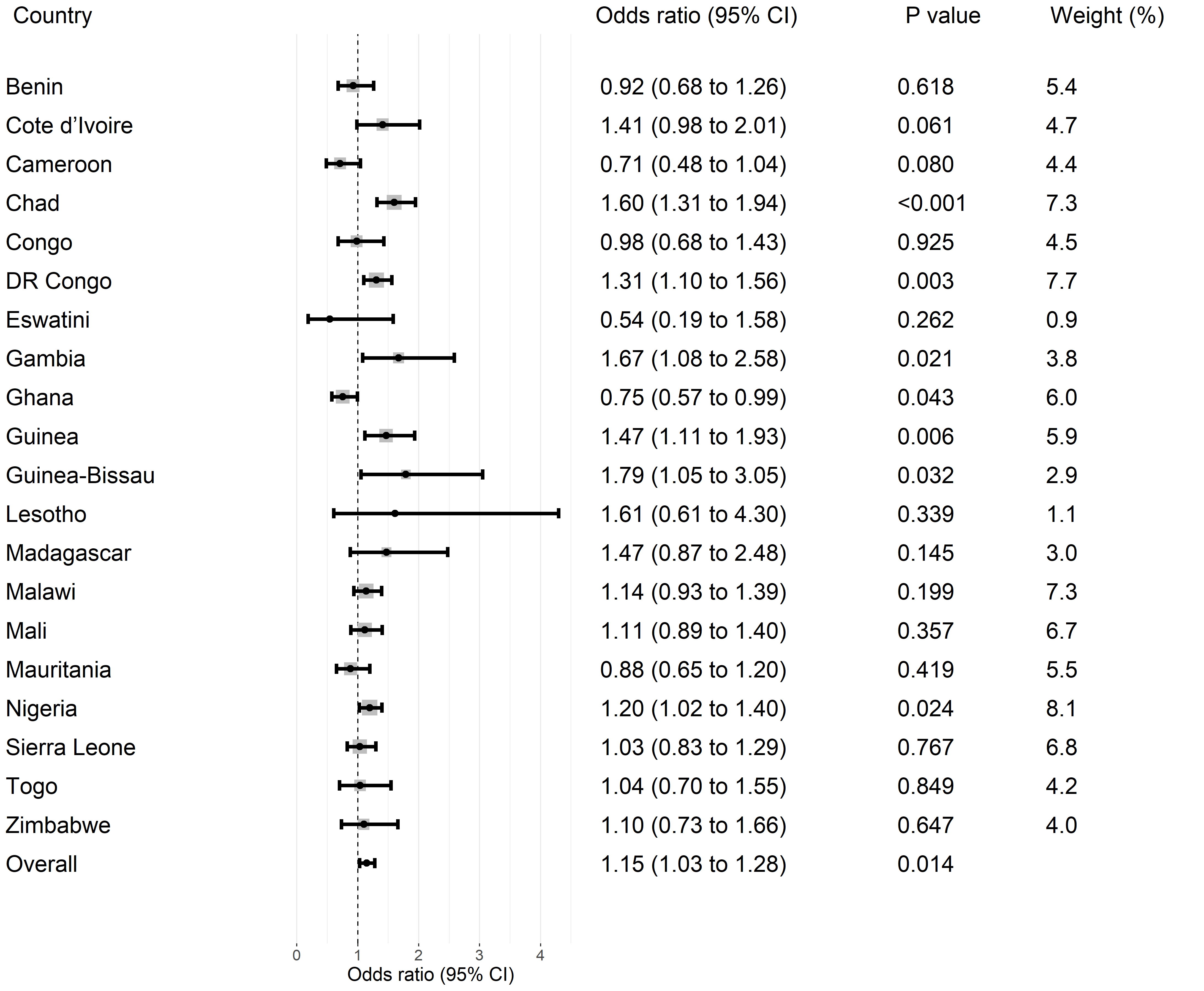
**Figure E.** Association between improved sanitation facilities and on-track cognitive development in children aged 36 to 59 months in sub-Saharan Africa.

CI: confidence interval; The weight given to each country is the inverse of the variance of the odds ratio estimate. Weight (%), which is indicated by the size of the box, is the proportion of the country’s weight that contributes to the sum of weights of all countries.


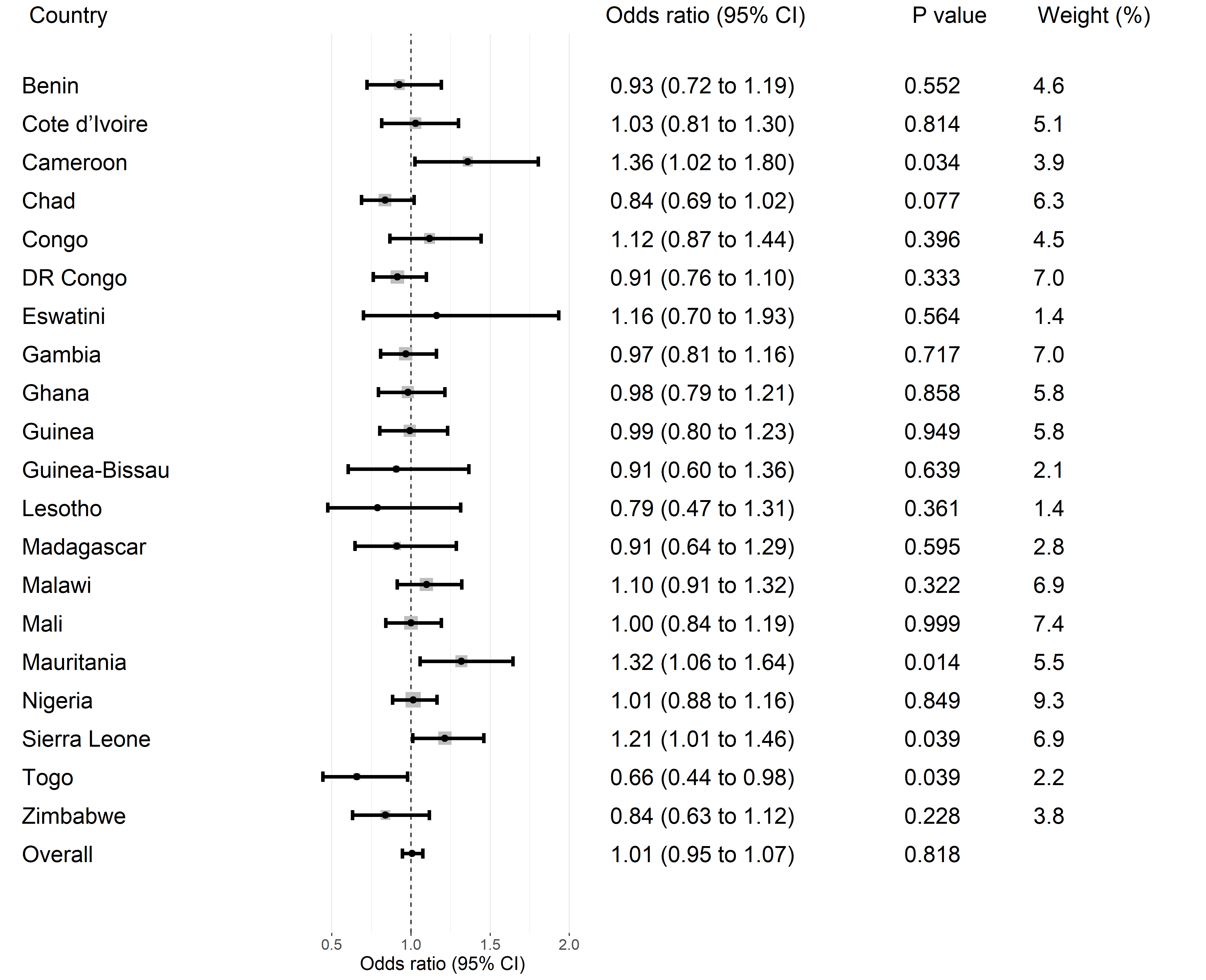
**Figure F.** Association between improved sanitation facilities and on-track social-emotional development in children aged 36 to 59 months in sub-Saharan Africa.

CI: confidence interval; The weight given to each country is the inverse of the variance of the odds ratio estimate. Weight (%), which is indicated by the size of the box, is the proportion of the country’s weight that contributes to the sum of weights of all countries.


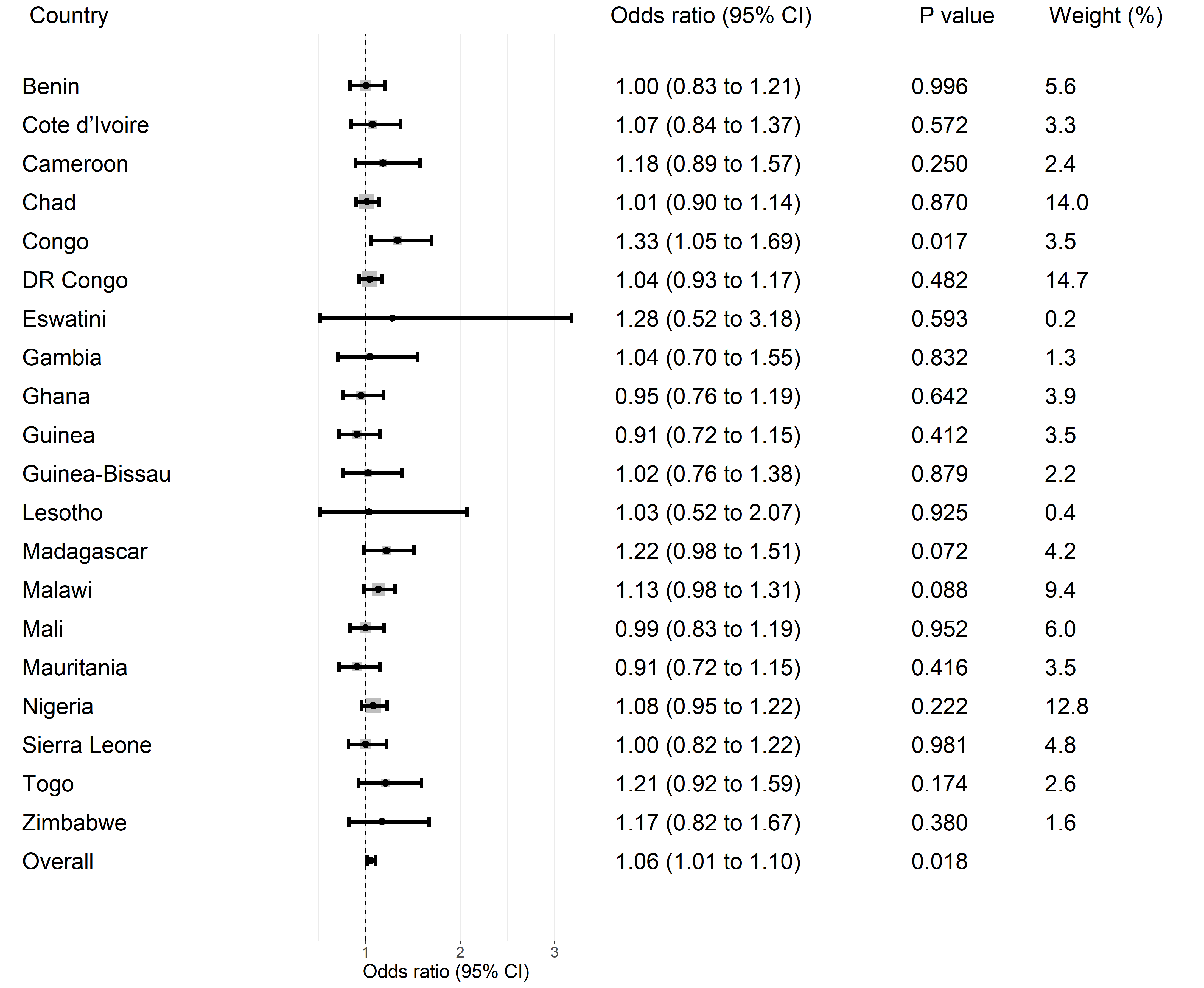
**Figure G.** Association between sufficient living area and on-track cognitive development in children aged 36 to 59 months in sub-Saharan Africa.

CI: confidence interval; The weight given to each country is the inverse of the variance of the odds ratio estimate. Weight (%), which is indicated by the size of the box, is the proportion of the country’s weight that contributes to the sum of weights of all countries.


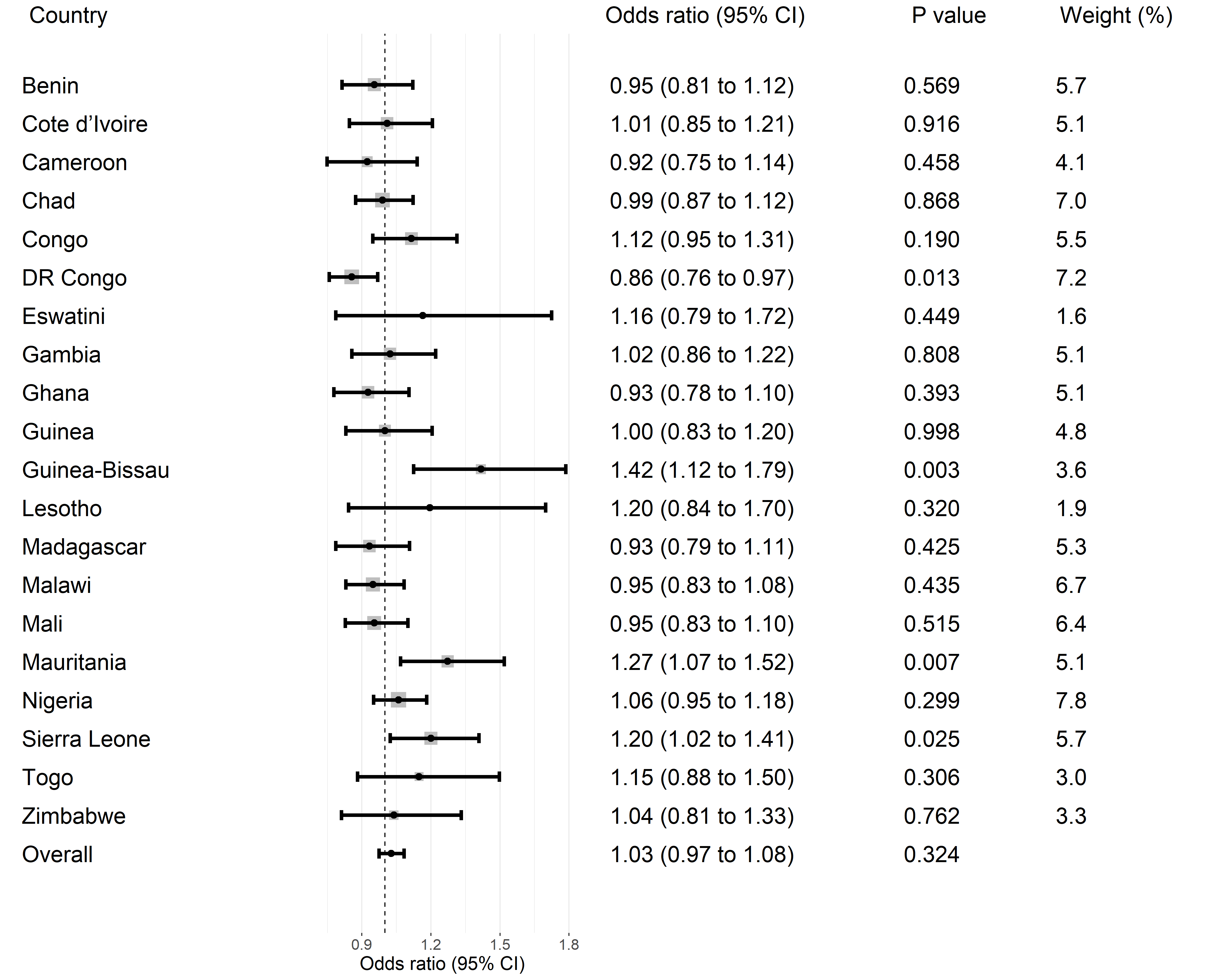
**Figure H.** Association between sufficient living area and on-track social-emotional development in children aged 36 to 59 months in sub-Saharan Africa.

CI: confidence interval; The weight given to each country is the inverse of the variance of the odds ratio estimate. Weight (%), which is indicated by the size of the box, is the proportion of the country’s weight that contributes to the sum of weights of all countries.


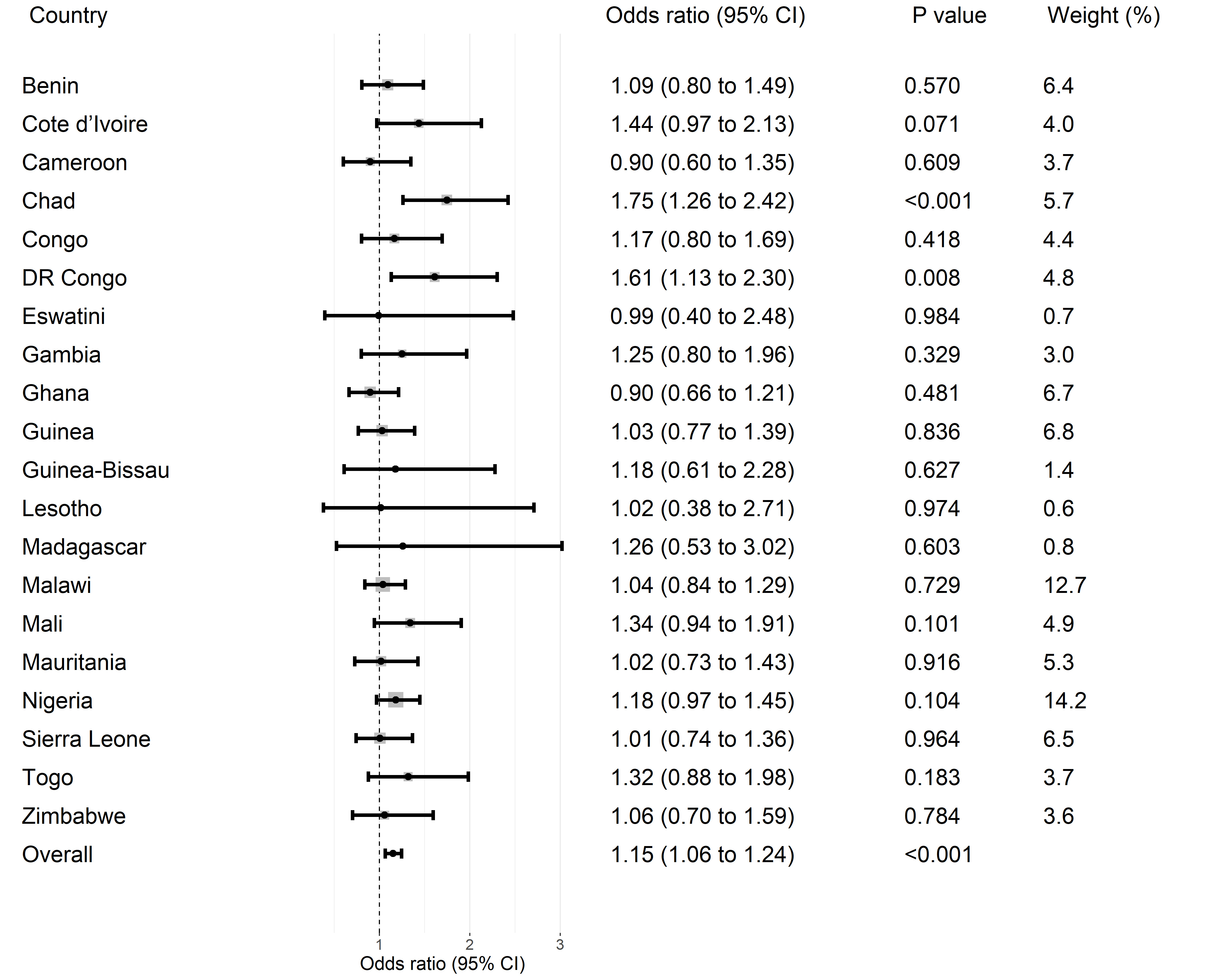
**Figure I.** Association between improved housing and on-track cognitive development in children aged 36 to 59 months in sub-Saharan Africa.

CI: confidence interval; The weight given to each country is the inverse of the variance of the odds ratio estimate. Weight (%), which is indicated by the size of the box, is the proportion of the country’s weight that contributes to the sum of weights of all countries.


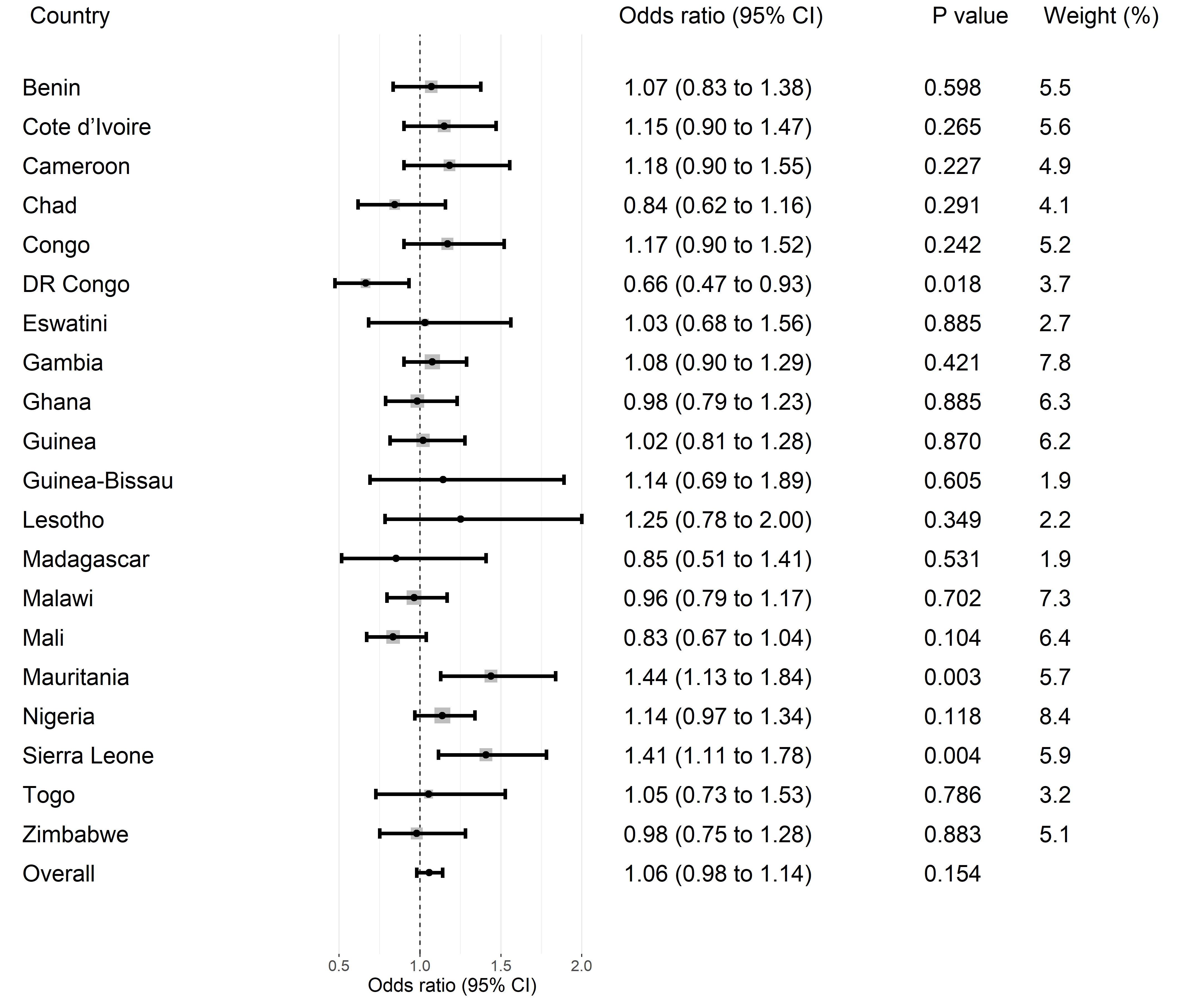
**Figure J.** Association between improved housing and on-track social-emotional development score in children aged 36 to 59 months in sub-Saharan Africa.

CI: confidence interval; The weight given to each country is the inverse of the variance of the odds ratio estimate. Weight (%), which is indicated by the size of the box, is the proportion of the country’s weight that contributes to the sum of weights of all countries.
