# Supplementary material for: Housing environment and early childhood development in sub-Saharan Africa: A cross-sectional analysis
Source: PLoS Med. 2021 Apr 19;18(4):e1003578. doi: 10.1371/journal.pmed.1003578 (PMC8092764; doi:10.1371/journal.pmed.1003578)
Supplement: S1 Fig — ECD, early childhood development; SSA, sub-Saharan Africa. (DOCX) [file pmed.1003578.s006.docx]

**S1 Fig.** Unadjusted association between housing quality and early childhood development in children aged 36 to 59 months in sub-Saharan Africa.


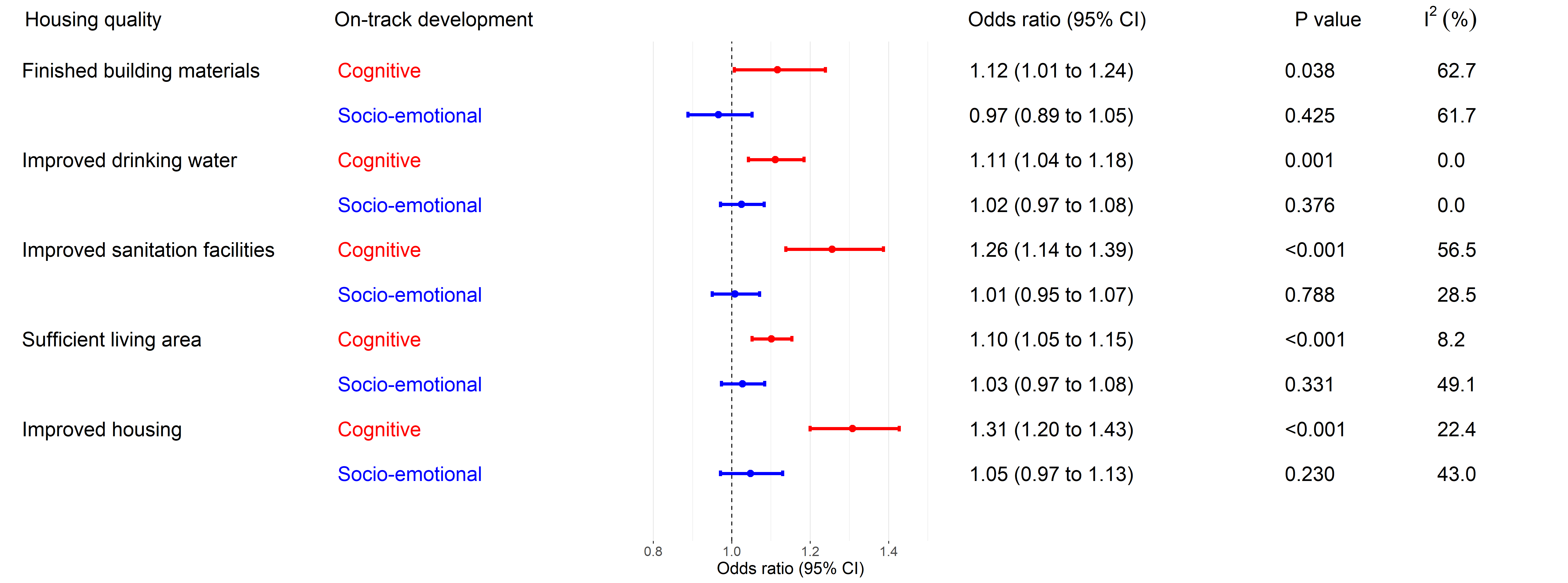


CI: confidence interval
