# Supplementary material for: Housing environment and early childhood development in sub-Saharan Africa: A cross-sectional analysis
Source: PLoS Med. 2021 Apr 19;18(4):e1003578. doi: 10.1371/journal.pmed.1003578 (PMC8092764; doi:10.1371/journal.pmed.1003578)
Supplement: S2 Fig — ECD, early childhood development; SSA, sub-Saharan Africa. (DOCX) [file pmed.1003578.s007.docx]

**S2 Fig.** Mutually adjusted association between housing quality and early childhood development in children aged 36 to 59 months in sub-Saharan Africa (adjusted for age (months) and gender of the child, maternal education, household wealth index, the availability of children’s books and playthings, and the other three characteristics of an improved house).


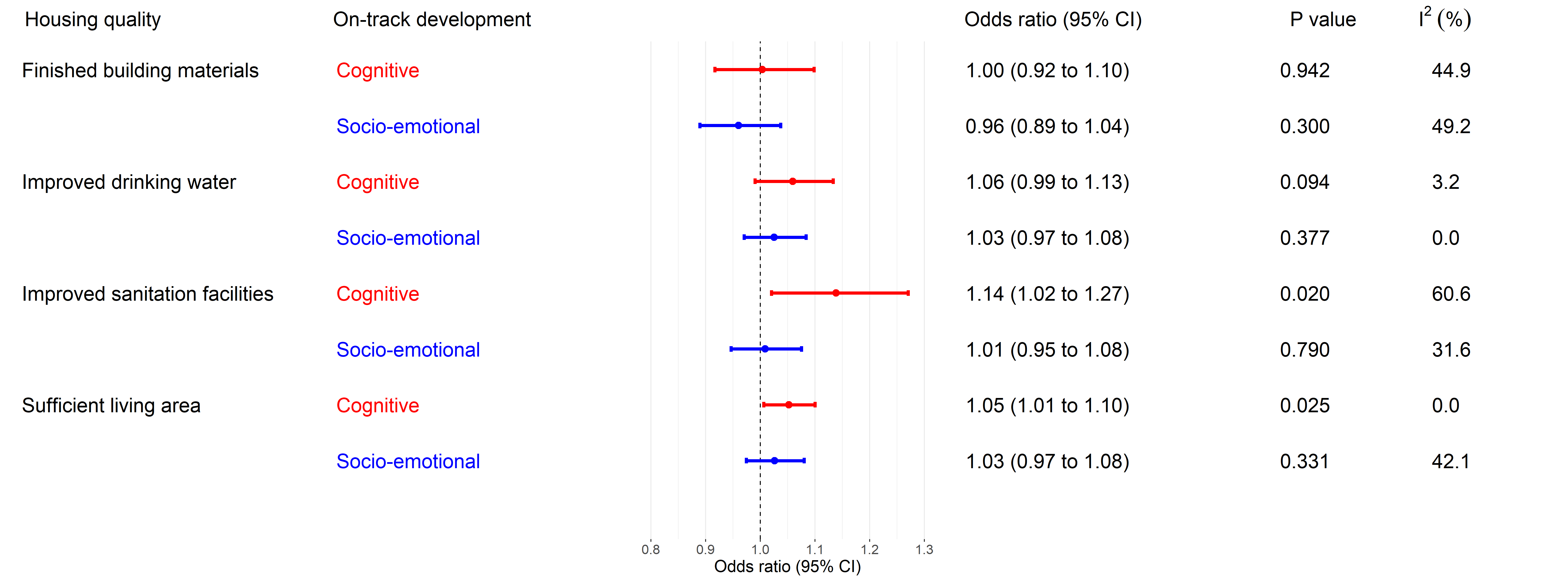


CI: confidence interval
